# TROP2 confers resistance to oxidative stress-induced cancer cell death through YAP/HMOX1 signaling

**DOI:** 10.1186/s12967-026-07955-z

**Published:** 2026-03-11

**Authors:** Di Wu, Yibing Liu, Ting Zhu, Haisen Zhao, Wanqiao Cao, Xiu Guo, Hongwei Xia, Yonglei Liu

**Affiliations:** 1 Experimental Research Center, QingPu Branch of Zhongshan Hospital Affiliated to Fudan University, Shanghai, China; 2 Department of Pathology, QingPu Branch of Zhongshan Hospital Affiliated to Fudan University, Shanghai, China; 3 Department of Thoracic Surgery, QingPu Branch of Zhongshan Hospital Affiliated to Fudan University, Shanghai, China; 4 Department of Respiratory Medicine, QingPu Branch of Zhongshan Hospital Affiliated to Fudan University, Shanghai, China

## Abstract

**Background:**

Trophoblast cell surface antigen 2 (TROP2) is overexpressed in non-small cell lung cancer (NSCLC) and associated with poor prognosis, yet its role in ferroptosis—an iron-dependent form of regulated cell death—remains largely unknown. This study investigated the function and underlying mechanism of TROP2 in ferroptosis regulation in lung cancer.

**Methods:**

TROP2 expression was examined in NSCLC tissues and cell lines. Functional assays, including RNA sequencing, qRT-PCR, Western blot, flow cytometry, and transmission electron microscopy, were performed in lung cancer cells with TROP2 knockdown or overexpression. The involvement of the YAP1/HMOX1 axis was evaluated using luciferase reporter assays, immunofluorescence, and nuclear-cytoplasmic fractionation. An in vivo xenograft model was established to assess the effect of TROP2 inhibition combined with the ferroptosis inducer RSL3.

**Results:**

TROP2 was significantly upregulated in NSCLC tissues and correlated with worse patient survival. Knockdown of TROP2 sensitized cells to RSL3-induced ferroptosis, as evidenced by increased lipid peroxidation, reactive oxygen species, and malondialdehyde levels, along with reduced glutathione. Mechanistically, TROP2 inhibited nuclear translocation of YAP1, which consequently suppressed transcriptional activation of the key ferroptosis-promoting gene HMOX1. Rescue experiments confirmed that HMOX1 mediates the pro-ferroptotic effect of TROP2 knockdown. In vivo, TROP2 depletion synergized with RSL3 to suppress tumor growth and elevate ferroptosis markers.

**Conclusion:**

TROP2 confers ferroptosis resistance in NSCLC by inhibiting YAP1 nuclear translocation and subsequent HMOX1 transcription. Targeting TROP2 sensitizes lung cancer cells to ferroptosis inducers, revealing a novel TROP2/YAP1/HMOX1 regulatory axis with therapeutic potential for NSCLC treatment.

**Supplementary Information:**

The online version contains supplementary material available at 10.1186/s12967-026-07955-z.

## Introduction

Lung cancer is a malignant tumor with highest global mortality rate and the diagnosis and treatment are improved, deepening on basic research. However, the patient incidence and mortality rates remain high, there needs further exploration of fundamental mechanisms to identify novel therapeutic targets.

Ferroptosis, a form of iron-dependent cell death, is characterized by iron-mediated lipid peroxidation and distinct from other types of cell death. Its hallmark is the reaction of intracellular free iron-containing enzymes with oxygen and lipids containing polyunsaturated fatty acids (PUFA)-containing lipids, generating high levels of membrane lipid peroxides. This process is initiated by membrane lipid peroxidation, which leads to abnormal ion flux, organelle damage (notably mitochondrial shrinkage and increased membrane density), and ultimately cell swelling and rupture. Ferroptosis sensitivity is highly cell type-specific, which is governed by diverse network of regulatory molecules [1–3]. There are some key regulatory molecules involved in ferroptosis are identified, such as glutathione peroxidase 4 (GPX4), Solute Carrier Family 7 Member 11 (SLC7A11, also known as xCT), and acyl-CoA synthetase long-chain family member 4 (ACSL4) [1–3]. GPX4, a phospholipid hydroperoxide glutathione peroxidase, protects cells by safeguarding membrane integrity [4]. SLC7A11, a cystine/glutamate antiporter, mediates amino acid transport across the plasma membrane [5]. ACSL4 catalyzes the activation of arachidonic acid (AA) to AA-CoA, facilitating its incorporation into membrane phospholipids [6]. Other recognized ferroptosis markers include HMOX1, FSP1, GCLC, and GCLM [1–3]. Given that cancer cells accumulate high iron levels compared to normal cells and ferroptosis inhibits tumor growth, elucidating its molecular mechanisms holds significant therapeutic potential.

Trophoblast cell surface antigen-2 (TROP2), a transmembrane glycoprotein, first identified in human choriocarcinoma cell lines, is encoded by the intronless *TACSTD2* gene located on chromosome 1p32 [7]. The TROP2 protein consists of 323 amino acids with an approximate molecular weight of 35kDa [7]. It is predominantly expressed in epithelial cells and plays notable roles in the embryonic and fetal development [7, 8]. While TROP2 expression remains low under normal physiological conditions, it is upregulated in a wide range of malignancies [9]. Reported expression rates include approximately 80% in breast cancer, 64% in lung adenocarcinoma, 75% in lung squamous cell carcinoma, 58.6% in epithelial ovarian cancer, and 71.8% in endometrioid carcinoma. Elevated TROP2 levels are associated with enhanced cellular survival, metastasis, and drug resistance [10, 11], highlighting its critical role in tumorigenesis and progression. Although research into cancer biology and some pathological mechanisms of TROP2 is ongoing, further investigation is required to fully elucidate its functions in cancer progression.

The Hippo pathway is a highly conserved signaling cascade that regulates critical biological processes including tissue growth, homeostasis maintenance, immune function, cancer development, and regeneration [12]. Through sequential kinase phosphorylation, it ultimately controls the activity of downstream transcriptional co-activators YAP and TAZ, key regulators of organ size [13]. As a core effector of Hippo pathway, YAP, participates in diverse physiological and pathological processes [13, 14] and plays a complex role in ferroptosis. On one hand, YAP can negatively regulate ferroptosis by inducing SLC7A11 expression in a TEAD- and ATF4- dependent manner, enabling cells to resist sorafenib induced ferroptosis [15]. On the other hand, YAP has been shown to promote ferroptosis in breast and colorectal cancer cells through upregulating modulators like ACSL4 and transferrin receptor 1 (TFRC) [16]. Nevertheless, the precise mechanisms governing YAP signaling in the regulation of ferroptosis remain incompletely understood.

Given TROP2 plays important roles in tumors, it is necessary to explore its new roles and mechanisms. Our preliminary research indicated that TROP2 suppression induces ferroptosis of lung cancer cells. TROP2 suppression led to ferroptosis and greatly increases HMOX1 levels in lung cancer cells, suggesting that TROP2 regulates ferroptosis via HMOX1. This study demonstrates that TROP2 modulates ferroptosis in lung cancer cells through the Hippo/YAP pathway.

## Materials and methods

### Tissue collection and ethics statement

Human lung cancer tissues were obtained from patients diagnosed with lung cancer at Qingpu Hospital (Fudan University, China) from December 2020 to August 2022. This study was approved by the Hospital Ethics Committee (Approved No: 2024-70). Written informed consent was obtained from participants prior to sample collection.

### Cell culture

Normal lung epithelial cells (BEAS-2B) and lung cancer cells (PC9, NCI-H1299, NCI-H292, NCI-H226, A549, Calu-1 and ChaGO-K-1) were obtained from Cell Bank of Chinese Academy of Science (Shanghai, China). Cells were cultured in RPMI-1640 medium supplemented with 10% fetal bovine serum and 100ng/ml penicillin-streptomycin and 37 °C in a 5% CO_2_ humidified incubator. Medium was changed every 3–5 days. For sphere formation assays, PC9 and NCI-H292 (H292 is used in the study) cells were cultured in DMEM/F12 with B27, EGF(10ng/ml) and FGF(10ng/ml). Spheres were imaged under an inverted microscope after 10–14 days of culture.

### Generation of overexpression and knockdown cells

The plasmids and siRNAs were transfected using Lipofectamine 2000 (Invitrogen, CA, USA) according to the manufacturer’s protocol. *TROP2*-, *HMOX1*- and *YAP1*-targeting siRNAs (sequences in Supplementary Table S1) were synthesized by Hanheng Biotechnology (Shanghai, China). Full-length *TROP2*, *YAP1*, and *HMOX1* cDNAs were cloned into pLKO.1-puro lentiviral vector (Fenghui Biotechnology, Shanghai, China). Lentivirus was packaged in HEK293T cells co-transfected with lentiviral vectors and packaging plasmids using Lipofectamine 2000. Infected cells with lentivirus were selected with 1 µg/mL puromycin for 48–72 h. Transduction efficiency was validated by western blotting.

### RNA sequencing

Total RNA was isolated and purified using TRIzol reagent (Invitrogen, Carlsbad, CA, USA) following the manufacturer’s procedure. The RNA amount and purity of each sample was quantified using NanoDrop ND-1000 (NanoDrop, Wilmington, DE, USA). The RNA integrity was assessed by Bioanalyzer 2100 (Agilent, CA, USA). Poly (A) RNA was purified from 1 µg total RNA using Dynabeads Oligo (dT) 25-61005 (Thermo Fisher, CA, USA) and fragmented into small pieces using Magnesium RNA Fragmentation Module (NEB, USA) under 94℃ 5–7 min. The cleaved RNA was reverse-transcribed to cDNA by SuperScript™ II Reverse Transcriptase (Invitrogen, cat896649, USA), which was used to synthesize U-labeled second-stranded DNAs using DNA polymerase I (NEB, USA), RNase H (NEB, USA) and dUTP (Thermo Fisher, USA). A-base was then added to the blunt ends of each strand. Each adapter contained a T-base overhang for ligating the adapter to the A-tailed fragmented DNA. Single- or dual-index adapters were ligated to the fragments, and size selection was performed with AMPureXP beads. After the heat-labile UDG enzyme (NEB, USA) treatment of the U-labeled second-stranded DNAs, the ligated products were amplified with PCR by the following conditions: initial denaturation at 95℃ for 3 min; 8 cycles of denaturation at 98℃ for 15 s, annealing at 60℃ for 15 s, and extension at 72℃ for 30 s; and then final extension at 72℃ for 5 min. The average insert size for the final cDNA library was 300 ± 50 bp. At last, we performed the 2 × 150 bp paired-end sequencing (PE150) on an illumine Novaseq™ 6000 following the vendor’s recommended protocol.

### Bioinformatics analysis of RNA-seq

A Fastp software (https://github.com/OpenGene/fastp) was used to remove the reads that contained adaptor contamination, low quality bases and undetermined bases with default parameter. Then sequence quality was also verified using fastp. HISAT2 (https://ccb.jhu.edu/software/hisat2) was used to map reads to the reference genome of Homo sapiens GRCh38. The mapped reads were assembled using String Tie (https://ccb.jhu.edu/software/stringtie) with default parameters. Then, all transcriptomes from all samples were merged to reconstruct a comprehensive transcriptome using gff compare (https://github.com/gpertea/gffcompare/). After the final transcriptome was generated, String Tie and was used to estimate the expression levels of all transcripts. Differential Expression: Adjusted p-value (FDR) < 0.05 and |log2(fold change)| > 1. Batch Correction: The ComBat function from the sva R package was applied to remove potential batch effects. Pathway Enrichment: GSEA was performed using the Hallmark and KEGG gene sets from MSigDB, with significance assessed by FDR < 0.25 as recommended.

### Quantitative real-time polymerase chain reaction

The RNA-quick purification kit (ES Science, Shanghai, China) was used to isolate total RNA from cells. Then, Color Reverse Transcription kit (EZ Bioscience, USA) was used to obtain cDNA. Genes expression were measured by qPCR system with 2×SYBR Green qPCR Master Mix (EZBioscience, USA). The primer sequences were listed in Supplementary Table S2.

### Western blotting

Lung cancer cells were seeded in 6-well plates with 1.5*10^6^ cells per well and subjected to indicated treatments. RIPA lysis buffer was used to extract total protein. Total protein (20-30ug/sample) was separated on SDS-PAGE (10%) and transferred to a PVDF membrane (Bio-Rad, Hercules, CA, USA). After that, the membranes were blocked in 5% fat-free milk for 1 h. The membranes were incubated with the primary antibodies including TROP2 (1:1000, cat #47866, CST), GPX4 (1:1000, cat #AF7020, Beyotime), SLC7A11 (1:1000, cat #AF7992, Beyotime), ACSL4 (1:1000, cat #AG1908, Beyotime), HMOX1 (1:1000, cat #AG2181, Beyotime), YAP1 (1:1000, cat #14074, CST), P-YAP1 (1:1000, cat #13619, CST), LATS1 (1:1000, cat #3477, CST), p-LATS1 (1:1000, cat #9157, CST), Histone3 (1:1000, cat #9715, CST),α-Tubulin (1:1000, cat #2144, CST) at 4 °C overnight and the secondary antibodies at room temperature for 2 h. Protein bands were visualized using the ECL (Bio-Rad, Hercules, CA, USA).

### Cell proliferation assay

CCK8 assay (Beyotime Biotechnology, Shanghai, China) was used for cell survival detection based on the manufacturer’s manual. Briefly, lung cancer cells in 100 µl culture medium were added into each well of a 96-well plate and subjected to special treatments. At each time point, 10ul of CCK8 was added to each well and incubated for 2 h at 37 °C. The absorbance at 450 nm was determined using a microplate reader. For colony formation assay, 1000–2000 cells were added to each well of a 6-well plate. The culture medium was replaced regularly. The cells were incubated for 10–14 days and the cellular colonies were formed. The plate was washed with 1×PBS gently and then the colonies were stained with 0.1% crystal violet. Finally the photos of the colonies in every well were taken.

### ROS and lipid peroxidation assay

The intracellular ROS level was determined using the ROS Detection Kit (Servicebio, G1706, Wuhan, China). The cells were seeded in 6-well plates, and the cells were treated with drugs for 24 h to ensure that the cell density was 50% − 70% during the detection. After the cells were digested and collected, they were washed 1–2 times with PBS buffer to reduce the interference of substances such as trypsin on the experimental results. The DCFH-DA probe was diluted with serum-free cell culture medium at a ratio of 1:1000 to prepare the working solution. 1 ml of working solution was added to make the cells evenly suspended in the centrifuge tube, and the cells were incubated in a 37 °C CO₂ incubator for 30 min in the dark. After the incubation, the cells were centrifuged at 800 g for 3–5 min to remove the working solution, and then washed 2–3 times with PBS buffer to fully remove the excess probe. Finally, the cells were resuspended in PBS. Quantification of lipid peroxidation by flow cytometry using C11-BODIPY 581/591 staining. The levels of ROS and lipid peroxidation were analyzed by flow cytometry (BD FACSAria Ⅲ).

### Malondialdehyde (MDA) assay

The determination of lipid peroxidation MDA was performed using the MDA Assay Kit (Servicebio, G4300, Wuhan, China). The samples were processed based on the kit instructions. The cells with indicated treatments were collected and suspended in PBS every ten million cells. The cells were dealted with ultrasonic and then centrifuge at 10,000 g for 15 min. The supernatant was taken for MDA detection. The samples were heated at 95 °C for one hour, cooled on ice and centrifuged at 10,000 g for 10 min. The absorbance at 532 nm were read using a microplate reader (Thermo Fisher Scientific, Waltham, MA, USA). Finally, total protein and MDA concentration of the samples were measured and data were analyzed (MDA concentration/protein concentration).

### Glutathione (GSH) assay

The level of glutathione (GSH) was determined using the GSH Detection Kit (Servicebio, G4305, Wuhan, China). After the cells were collected, they were suspended in PBS by adding 300 µL of PBS to every one million cells. Ultrasonication was conducted at 4 °C with a frequency of 25 kHz, involving 30 s of sonication and 20 s of interval, for a total duration of 30 min. Subsequently, the cells were centrifuged at 8000 g for 10 min. The supernatant was collected, mixed with the protein removal reagent, and centrifuged once again. The supernatant was obtained for the subsequent detection of reduced glutathione. The samples were mixed with the working solution of the detection probe and added to a 96-well plate. After incubation at room temperature for 5 min, the absorbance at 412 nm was detected using a Multifunctional Microplate Reader (Thermo Fisher Scientific, Waltham, MA, USA). The concentration of GSH was correlated with the absorbance and calculated via the standard curve.

### Transmission electron microscopy (TEM) analysis

The cells with TROP2 downregulation combined with or without RSL3 treatment were collected and fixed with 4% paraformaldehyde for 24 h, and then fixed in 1% osmium acid prepared in 0.1 M phosphate buffer PB (PH7.4) at room temperature in the dark for 2 h. After dehydration with an ethanol gradient, the samples were infiltrated and embedded. Cut 1.5 μm semi-thin sections of the resin block with a semi-thin sectioning machine were down, stained with toluidine blue, and located them under a light microscope. Finally, the slices were observed under a transmission electron microscope (HITACHI, HT7800).

### Nuclear and cytoplasmic fractionation

To separate nuclear and cytoplasmic proteins, cells were fractionated using the Nuclear and Cytoplasmic Protein Extraction Kit (Beyotime, P0027, Beijing, China). Cells were collected by scraping, washed with PBS, and pelleted by centrifugation at 500 g for 5 min. The cell pellet was then suspended in cytoplasmic extraction reagent supplemented with protease inhibitors, incubated on ice, and centrifuged at 12,000 g for 10 min to obtain the supernatant (cytoplasmic fraction). The remaining pellet was washed and suspended in nuclear extraction reagent to isolate the nuclear proteins. Fractionation efficiency was confirmed by probing for the cytoplasmic marker GAPDH/Tubulin and the nuclear marker Histone H3 via Western blotting.

### Immunofluorescence

For immunofluorescence staining, cells were seeded onto a glass bottomed 24 well plate (Cellvis, P24-1.5 H-N) and subjected to special treatments. Then, cells were fixed with 4% paraformaldehyde at room temperature for 15 min, permeabilized with 0.1% Triton X100, and blocked with 1% BSA at room temperature for 1 h. Subsequently, cells were incubated with the primary antibodies against YAP1, HMOX1 overnight at 4℃. The following day, cells were incubated with appropriate fluorescent dye-conjugated secondary antibodies (anti-rabbit or anti-mouse) at room temperature for 1 h. Nuclei were counter-stained with DAPI for 15 min. The images were taken under an Olympus FV3000 confocal microscope.

### Hematoxylin and eosin (HE) and immunohistochemistry (IHC)

The paraffin-embedded tumor tissues were sectioned at a thickness of 5 μm. Following deparaffinization and rehydration through a graded ethanol series, sections were strained with hematoxylin-eosin staining (HE) according to standard protocols. After straining, sections were dehydrated with alcohol, cleared in xylene, and then mounted with neutral resin. The imagines were acquired using a microscope (Olympus, CKX53, Tokyo, Japan).

For IHC, deparaffinized and rehydrated tissue sections underwent antigen retrieval in citrate buffer (pH 6.0) using a microwave heating method. Endogenous peroxidase activity was quenched with 3% H₂O₂ for 15 min. After blocking with 5% normal goat serum for 1 h at room temperature, sections were incubated overnight at 4 °C with primary antibodies (e.g., anti-TROP2, 1:100, anti-SLC7A11, 1:100, anti-HMOX1, 1:100). After washing, sections were incubated with a biotinylated secondary antibody (e.g., anti-rabbit IgG) for 1 h at room temperature, followed by the ABC reagent (Vector Laboratories). Diaminobenzidine (DAB) was used as the chromogen, and hematoxylin was applied for nuclear counterstaining. Sections were then dehydrated, cleared, and mounted for observation under an Olympus CKX53 microscope.

### Luciferase reporter assay

The putative promoter region of the human HMOX1 gene was amplified from genomic DNA. A series of 5‘-deletion constructs were generated by PCR, including the full-length promoter (e.g., -2000 to + 200 bp relative to the transcription start site) and progressively shorter fragments (e.g., -1000/+200, -500/+200, -200/+200 bp). All fragments were cloned into the pGL3-Basic vector (Promega). For transfection, cells (e.g., HEK293T) were seeded in 24-well plates and co-transfected with 400 ng of the respective pGL3-promoter construct, 100 ng of the Renilla luciferase control plasmid pRL-TK (Promega), and 500 ng of either a YAP expression plasmid or the corresponding empty vector, using Lipofectamine 3000 (Invitrogen) according to the manufacturer‘s protocol. After 48 h, firefly and Renilla luciferase activities were measured using the Dual-Luciferase Reporter Assay System (Promega). Firefly luciferase activity was normalized to Renilla activity for each well. The normalized activity for each construct in the presence of YAP is presented as fold activation relative to its activity with the empty vector control.

### Xenograft tumor model

All animal experiments were approved by the Committee of Animal Experimental Ethical Inspection of Fudan University (Approval number: 202406006 S) and conducted in accordance with institutional guidelines for the care and use of laboratory animals. Male BALB/c nude mice (4–6 weeks old) were used for the xenograft studies. PC9 cells stably expressing either control shRNA (shCON) or TROP2-targeting shRNA (shTROP2) were harvested and resuspended in cold PBS. Each mouse was subcutaneously injected with 1 × 10^7^ cells in a total volume of 100 µL into the right flank. When the tumor volumes reached approximately 100 mm³, the mice were randomly divided into four groups (*n* = 6 per group): shCON group, Intraperitoneal (i.p.) injection of vehicle, every other day; shCON + RSL3 group, i.p. injection of RSL3 (5 mg/kg), every other day; shTROP2 group, i.p. injection of vehicle, every other day; shTROP2 + RSL3 group, i.p. injection of RSL3 (5 mg/kg), every other day. The treatment schedule is illustrated in Fig. 7A. Tumor dimensions (length and width) were measured every two days with a caliper, and the volume was calculated using the formula: Volume (mm³) = (length × width²) / 2. Body weight was monitored simultaneously as an indicator of overall health. After 13 days of treatment, all mice were euthanized by cervical dislocation under anesthesia. Tumors were excised, weighed, and photographed. A portion of each tumor was fixed in 4% paraformaldehyde for immunohistochemistry (IHC) analysis, and the remainder was snap-frozen in liquid nitrogen for subsequent biochemical assays.

**Fig. 1:**
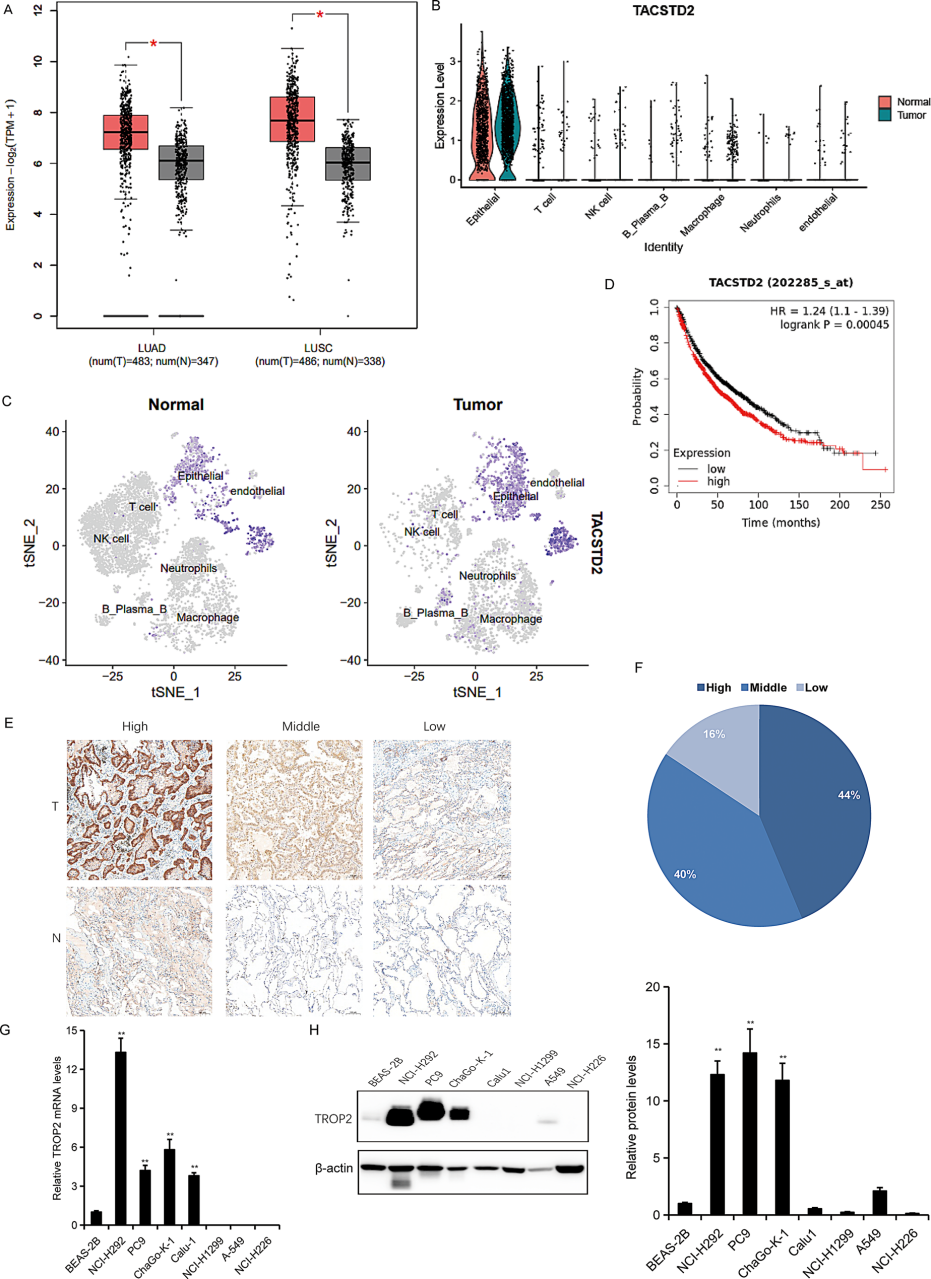
TROP2 is aberrantly overexpressed in lung cancer and correlates with poor prognosis. (**A**) Analysis of TROP2 expression in lung (adenocarcinoma) and lung squamous cell carcinoma (LUSC) tissues, based on data from cancer tissues from GEPIA database (cancer-pku.cn). **(B-C**) Expression of TROP2 in lung cancer tissues based on single-cell RNA sequencing (scRNA-seq) data. (**D**) Kaplan-Meier survival analysis showing the correlation between high TROP2 expression and poor prognosis in lung cancer patients. (**E**) Representative immunohistochemistry (IHC) images showing TROP2 expression in lung cancer tissues and paired adjacent normal tissues.(**F**) Quantitative summary of TROP2 IHC staining intensity in lung cancer tissues (*n* = 69). (**G**) TROP2 mRNA levels in the normal lung cell line BEAS-2B and in lung cancer cells (PC9, H1299, H292, H226, A549, Calu-1, and ChaGO-K-1). The cells were cultured and their total RNA was isolated for real time RT-PCR analysis. (**H**) TROP2 protein levels in BEAS-2B and the indicated lung cancer cells, as analyzed by western blot. Quantitative analysis (bar graph) of the bands was shown adjacent to the representative blots. Data are presented as mean ± SD (*n* = 3). Statistical significance was determined by one-way ANOVA followed by Dunnett’s post hoc test for comparisons against the BEAS-2B control group. ∗*P* < 0.05, ∗∗*P* < 0.01

**Fig. 2:**
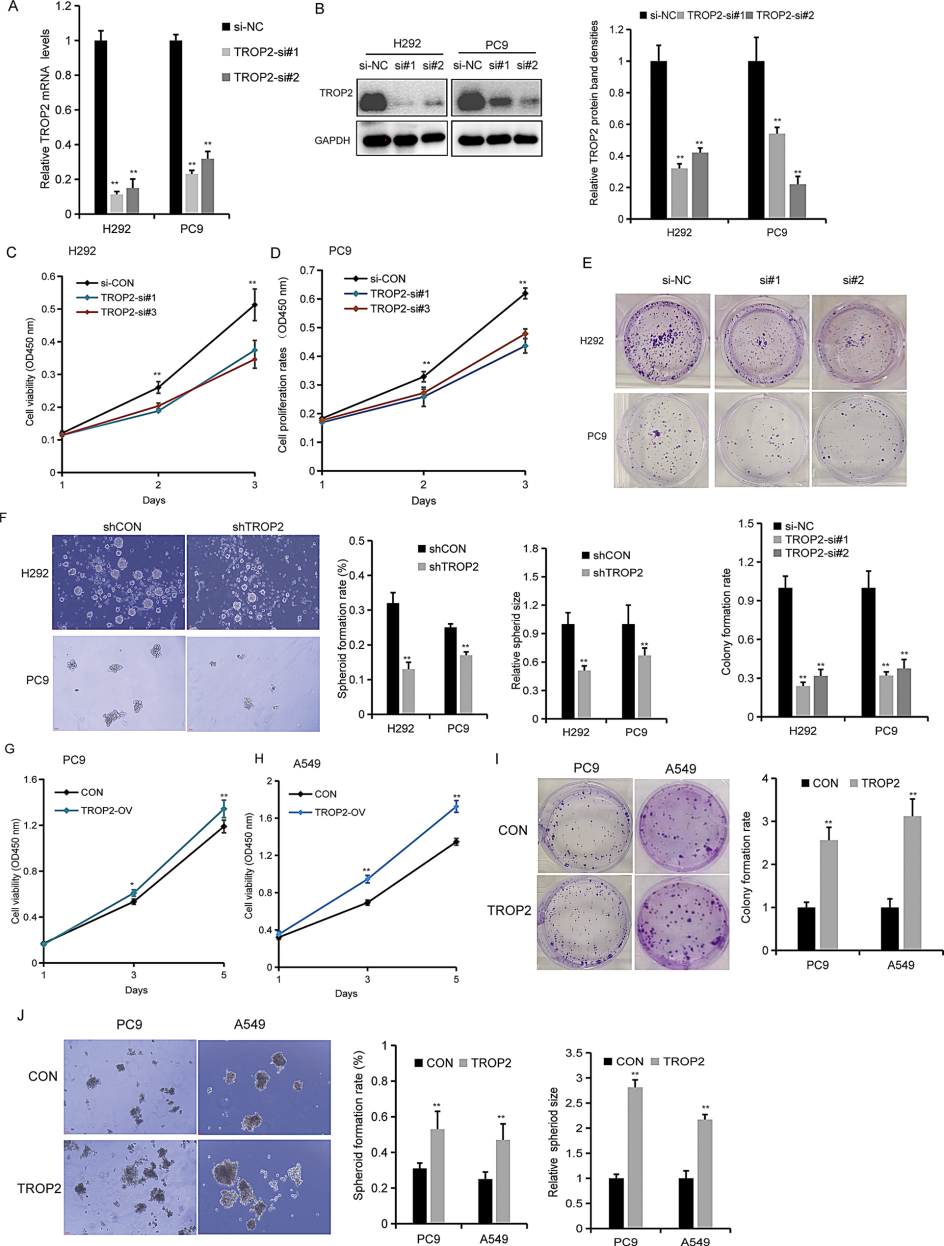
TROP2 regulates the malignant phenotypes of NSCLC cells. (**A, B**) Knockdown efficiency of TROP2 in PC9 and H292 cells transfected with TROP2-targeting siRNAs was confirmed at the mRNA level with qRT-PCR (**A**) and at the protein level by western blot (**B**). Quantitative analysis (bar graph) of the bands was shown adjacent to the representative blots. (**C, D**) The cell viability was suppressed in PC9 and H292 cells with TROP2 down-regulation. Cell proliferation was evaluated by CCK8. (**E**) The colonies were reduced in PC9 and H292 cells with TROP2 down-regulation using colony formation assay. (**F**) Represent spheres of lung cancer cells with TROP2 down-regulation. (**G, H**) The cell viability was assayed in PC9 and A549 cells with TROP2 overexpression by CCK8. (**I**) Colony formation assay was used to evaluate cell viability in PC9 and A549 cells with TROP2 over-expression. (**J**) Representative images of tumor spheres formed of lung cancer cells with TROP2 over-expression. Data are presented as mean ± SD (*n* = 3). Statistical significance was determined by two-tailed unpaired Student’s t-test (**A**, **B**, **E**, **F**, **I**, **J**) and two-way ANOVA followed by Sidak’s post hoc test (**C**, **D**, **G**, **H**). ∗*P* < 0.05, ∗∗*P* < 0.01

**Fig. 3:**
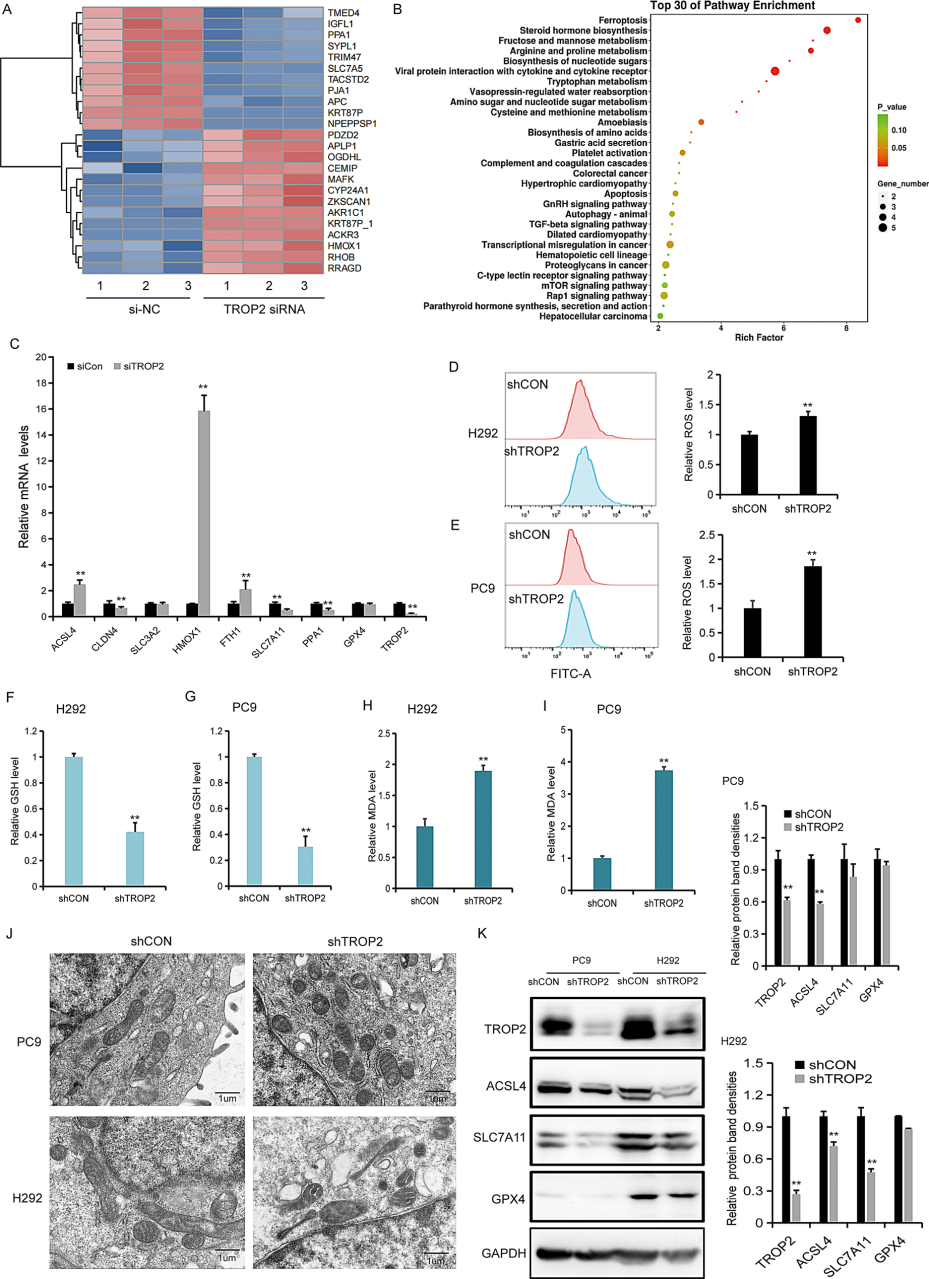
TROP2 inhibition potentiates ferroptosis in lung cancer cells. (**A**) Heatmap displaying the top 24 differentially expressed genes in TROP2-knockdown H292 cells compared to control. (**B**) KEGG pathway analysis revealed a significant association of the ferroptosis pathway with TROP2 knockdown. (**C**) Validation of mRNA expression levels of key ferroptosis-related genes (HMOX1, SLC7A11, ACSL4) by qRT-PCR in TROP2-knockdown cells. (**D, E**) Flow cytometric analysis of reactive oxygen species (ROS) levels in H292 and PC9 cells after TROP2 knockdown. (**F, G**) Measurement of intracellular GSH levels in H292 and PC9 cells with TROP2 knockdown. (**H, I**) Measurement of MDA levels in H292 and PC9 cells with TROP2 knockdown. (**J**) Representative transmission electron microscopy (TEM) images of H292 and PC9 cells after TROP2 knockdown, showing characteristic ferroptotic mitochondrial morphology (e.g., shrunken mitochondria with increased membrane density and diminished cristae). (**K**) Western blot analysis of TROP2 and key ferroptosis-related proteins (SLC7A11, GPX4, and ACSL4) in control and TROP2-knockdown H292 and PC9 cells.Quantitative analysis (bar graph) of the bands was shown adjacent to the representative blots. Data are presented as mean ± SD (*n* = 3). Statistical significance was determined by one-way ANOVA followed by Tukey’s post hoc test (**C**) and two-tailed unpaired Student’s t-test (**D**, **E**, **F**, **G**, **H**, **I**, **K**). ∗*P* < 0.05, ∗∗*P* < 0.01

**Fig. 4:**
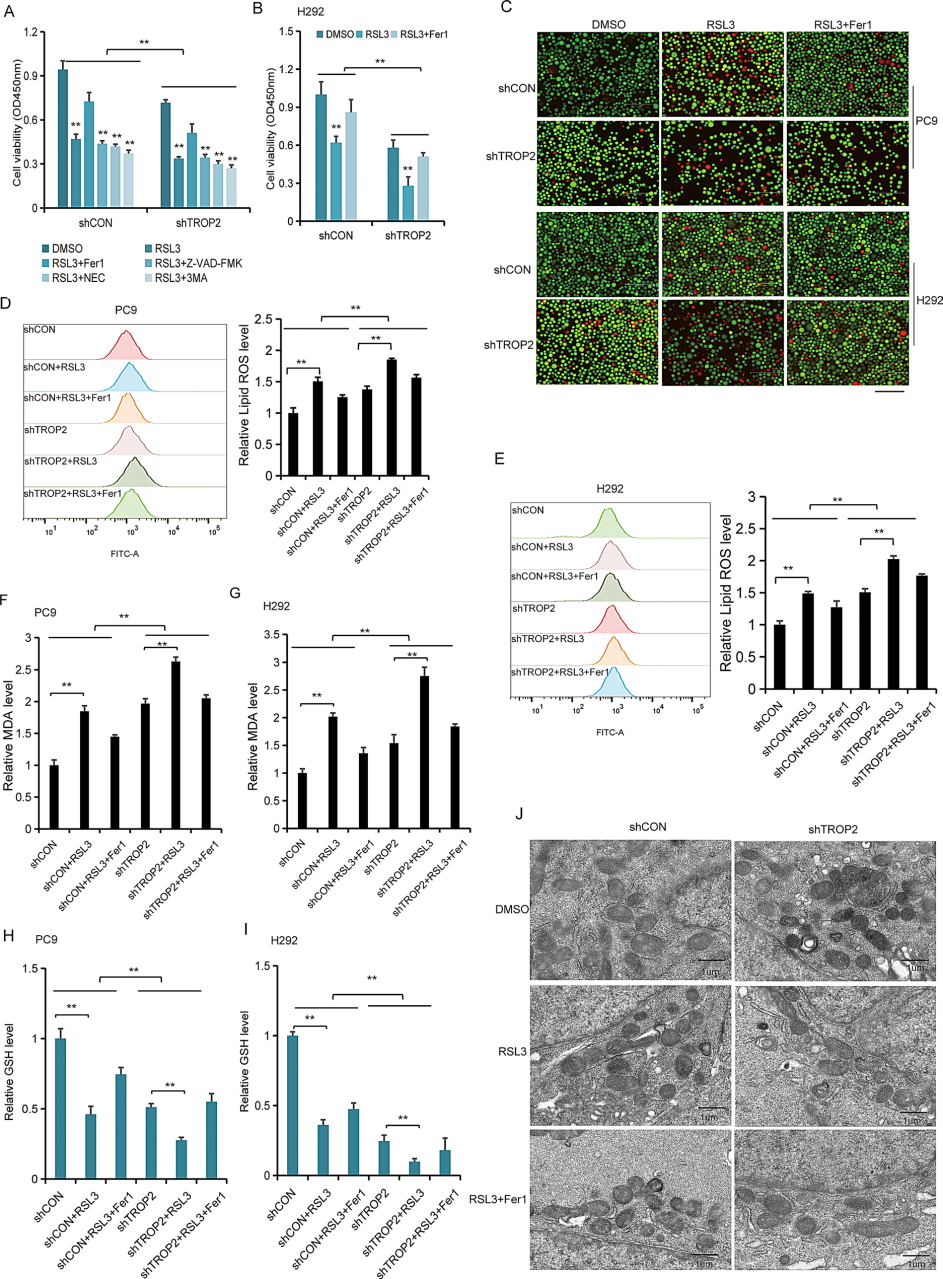
Inhibiting TROP2 expression enhances RSL3 induced ferroptosis in NSCLC cells. (**A**) Validation of ferroptosis-specific cell death. Viability of PC9 cells with TROP2 knockdown treated with RSL3, in the presence or absence of inhibitors targeting different cell death pathways: Ferrostatin-1 (Fer-1, ferroptosis, 5µM), Z-VAD-FMK (apoptosis, 20µM), Necrostatin-1 (necroptosis, 10µM), and 3-Methyladenine (3-MA, autophagy, 5 mM). Only Fer-1 significantly rescued cell death, confirming the ferroptosis-specific effect. **(B**) The cell viability of H292 cells with or without TROP2 knockdown, treated with RSL3, as determined using the CCK-8 assay. (**C**) Representative LIVE/DEAD staining images PC9 and H292 cells with TROP2 down-regulation in the presence of RSL3 (5 μm) or the ferroptosis inhibitor Ferrostatin-1 (Fer1, 5 μm). (**D, E**) Flow cytometric analysis of Lipid ROS levels were detected using C11-BODIPY staining in TROP2 knockdown cells with or without 5 μm RSL3 for 24 h. (**F, G**) The cellular MDA levels were measured in TROP2 knockdown cells with or without 5 μm RSL3 for 24 h. (**H, I**) The cellular GSH levels were measured in TROP2 knockdown cells with or without 5 μm RSL3 for 24 h. (**J**) Representative TEM images of H292 and PC9 cells with TROP2 down-regulation, further treated with RSL3 (5ug/ml), showing ferroptotic features. Data are shown as the mean ± SD (*n* = 3). Data are shown as the mean ± SD (*n* = 3). Statistical significance was determined by two-way ANOVA followed by Sidak’s post hoc test (**A**, **B**) and two-tailed unpaired Student’s t-test (**D**, **E**, **F**, **G**, **H**, **I**). ∗*P* < 0.05, ∗∗*P* < 0.01

**Fig. 5:**
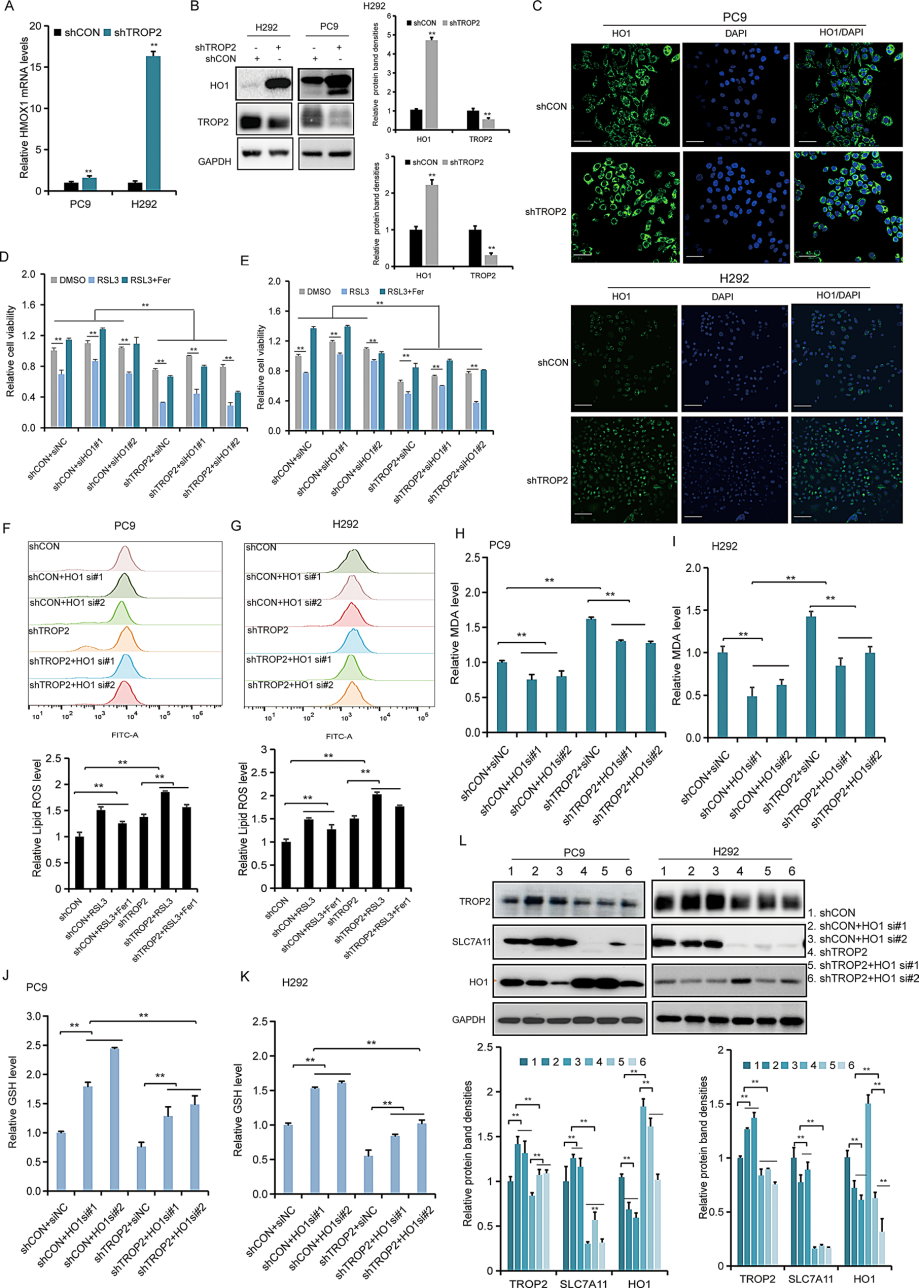
TROP2 suppresses ferroptosis by transcriptionally repressing HMOX1 in NSCLC cells. (**A**) HMOX1 mRNA levels in H292 and PC9 cells upon TROP2 knockdown, as determined by qRT-PCR.(**B**) HMOX1 protein levels in H292 and PC9 cells with TROP2 knockdown or overexpression using western blotting. Quantitative analysis (bar graph) of the bands was shown adjacent to the representative blots. (**C**) HMOX1 protein levels in PC9 and H292 cells with TROP2 down-regulation. HMOX1 protein levels were was examined by IF. (**D, E**) The cell viability of H292 and PC9 cells after TROP2 down-regulation was determined using the CCK-8 assay. H292 and PC9 cells subjected to TROP2 knockdown, with or without concomitant HMOX1 knockdown, and treated with RSL3. (**F, G**) Lipid peroxidation was detected using C11-BODIPY staining in TROP2 knockdown cells with or without 5 μm RSL3 for 24 h. (**H, I**) MDA levels were assayed in TROP2 knockdown cells with or without 10 μm RSL3 for 24 h. (**J, K**) GSH levels were assayed in TROP2 knockdown cells with or without 5 μm RSL3 for 24 h. (**L**) Western blot analysis of key ferroptosis associated proteins in PC9 and H292 cells under the indicated genetic and treatment conditions (TROP2 knockdown ± HMOX1 knockdown, with RSL3). Quantitative analysis (bar graph) of the bands was shown below to the representative blots. Data are shown as the mean ± SD (*n* = 3). Statistical significance was determined by two-tailed unpaired Student’s t-test (**A**); two-way ANOVA followed by Sidak’s post hoc test (**F**-**K**); three-way ANOVA followed by Sidak’s post hoc test (**D**, **E**). ∗*P* < 0.05, ∗∗*P* < 0.01

**Fig. 6:**
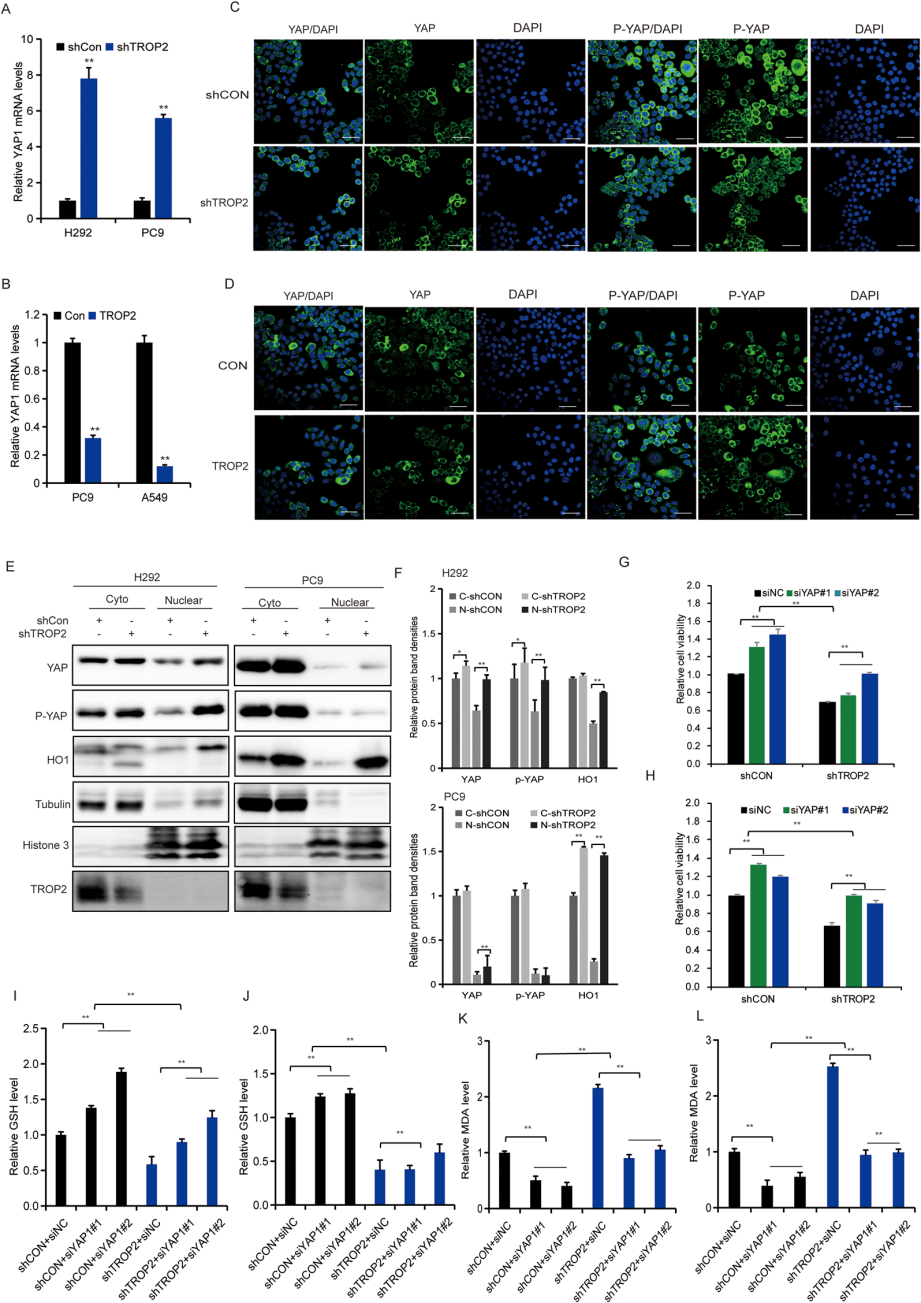
TROP2 confers ferroptosis resistance by inactivating YAP to suppress HMOX1 transcription. (**A**) YAP1 mRNA levels in H292 and PC9 cells with TROP2 knockdown, as determined by qRT-PCR. (**B**) YAP1 mRNA levels in PC9 and A549 cells with TROP2 overexpression, as determined by qRT-PCR. (**C**) Representative IF images showing enhanced nuclear localization of YAP (green) in PC9 cells upon TROP2 knockdown. Nuclei were stained with DAPI (blue). Scale bar, 50 μm. (**D**) Representative IF images showing suppressed nuclear localization of YAP (green) in PC9 cells upon TROP2 overexpression. Scale bar, 50 μm. (**E**) Western blot analysis of YAP protein levels in nuclear and cytoplasmic fractions of PC9 and H292 cells with or without TROP2 knockdown. (**F**) Quantitative analysis (bar graph) of the bands was shown adjacent to the representative blots (from E). (**G, H**) Cell viability assessed by CCK-8 assay in PC9 and H292 cells with TROP2 knockdown, with or without concomitant YAP inhibition, following RSL3 treatment. (**I,J**) Measurement of cellular GSH levels in PC9 and H292 cells under the indicated conditions. (**K, L**) Measurement of cellular MDA levels in PC9 and H292 cells under the indicated conditions. Data are presented as mean ± SD (*n* = 3). Statistical significance was determined by two-tailed unpaired Student’s t-test (**A**, **B**, **F**); two-way ANOVA followed by Sidak’s post hoc test or three-way ANOVA followed by Sidak’s post hoc test (**G**-**L**). ∗*P* < 0.05, ∗∗*P* < 0.01

**Fig. 7:**
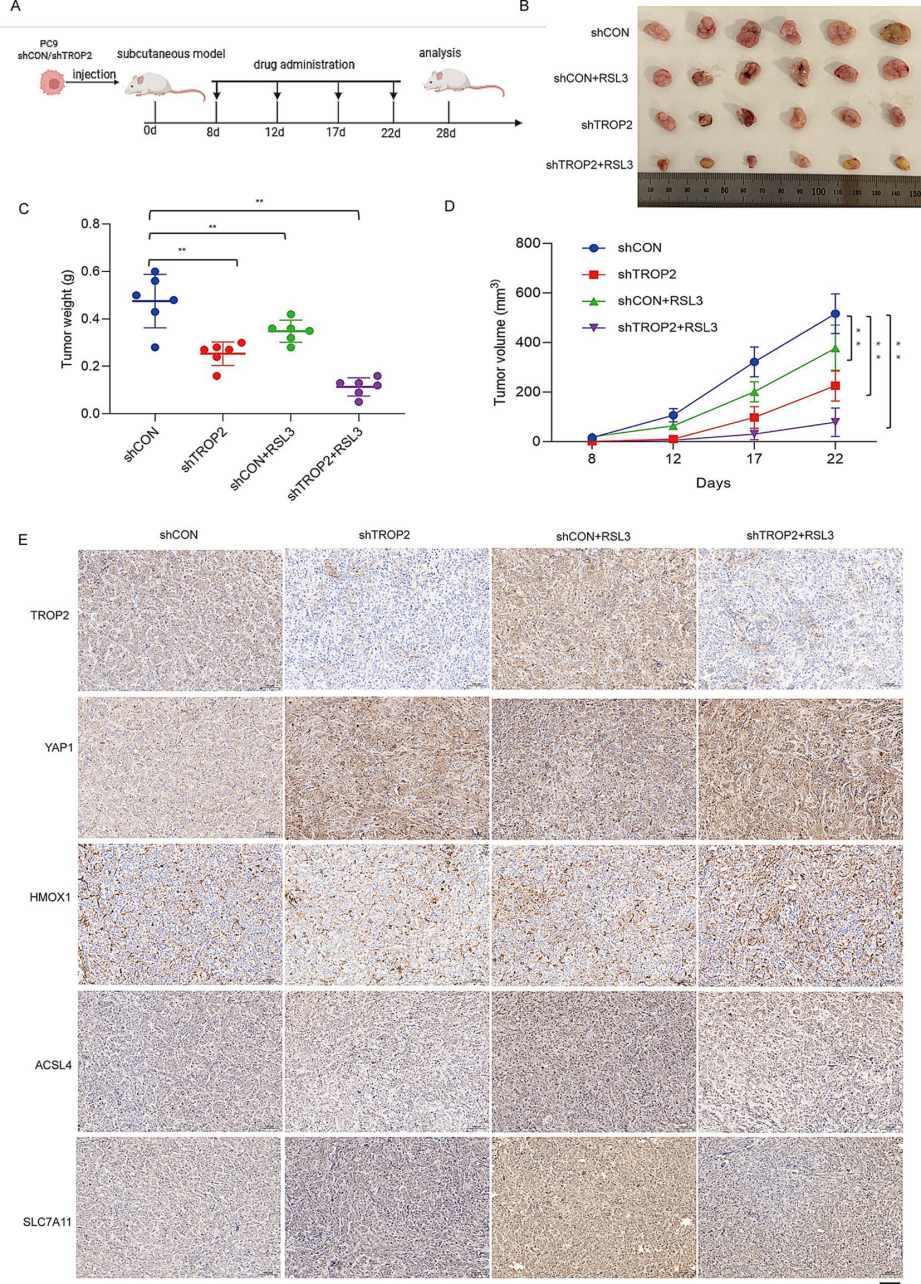
TROP2 suppression sensitized NSCLC to RSL3-induced ferroptosis in vivo. (**A**) Schematic diagram of the in vivo experimental timeline. Nude mice were subcutaneously injected with control (shCON) or TROP2-knockdown (shTROP2) PC9 cells, followed by intraperitoneal injection of RSL3 (5 mg/kg) or vehicle every other day. (**B**) Representative photographs of dissected subcutaneous tumors from each treatment group at the endpoint of the study. (**C**) Final tumor weights from each group at the endpoint. Data are presented as mean ± SD (*n* = 6). (**D**) Tumor growth curves showing tumor volume over time for the four experimental groups. Data are presented as mean ± SEM (*n* = 6 mice per group). (**E**) Representative IHC images of tumor sections stained for TROP2, YAP1, HMOX1, ACSL4, and SLC7A11 from the indicated groups. Scale bar, 100 μm. Data are presented as mean ± SD (*n* = 6, C) or mean ± SEM (*n* = 6 mice per group, **D**). Statistical significance for differences among groups was determined by one-way ANOVA followed by Tukey’s post hoc test (C, and for the final time point in D). ∗*P* < 0.05, ∗∗*P* < 0.01

**Fig. 8:**
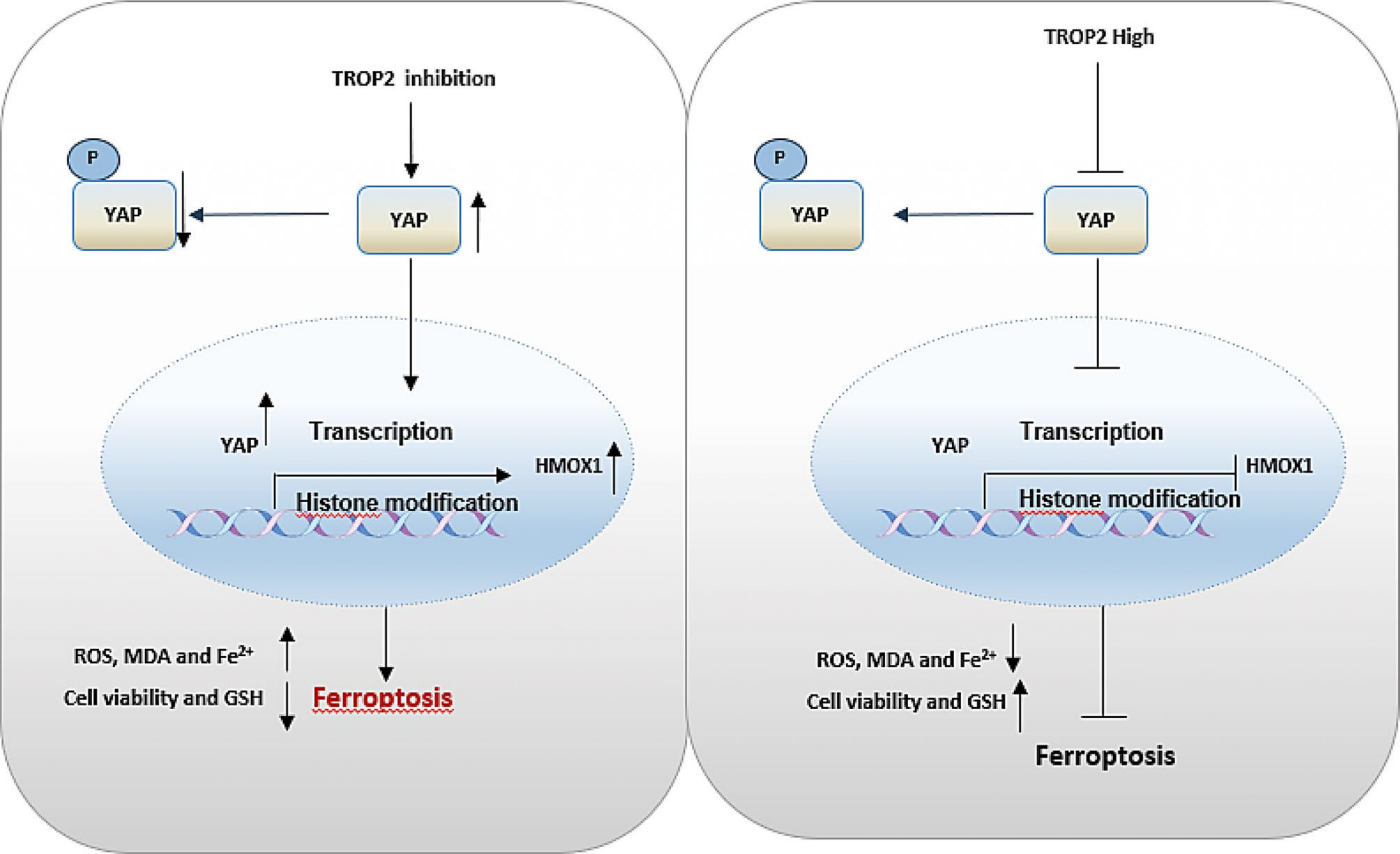
Working model of TROP2-mediated ferroptosis resistance in NSCLC. In NSCLC, high TROP2 expression promotes tumor progression by enhancing proliferation and survival. Mechanistically, TROP2 inhibits ferroptosis—an iron-dependent form of regulated cell death—by sequestering YAP in the cytoplasm, thereby suppressing its nuclear translocation and subsequent transcriptional activation of the pro-ferroptotic gene HMOX1. Targeted inhibition of TROP2 restores YAP/HMOX1 signaling, sensitizing tumors to ferroptosis inducers (e.g., RSL3), and supporting a potential therapeutic strategy for NSCLC

### Statistical analysis

The data were presented as the mean ± standard deviation (SD) taken from at least three independent experiments. The comparisons between groups were performed using Student’s t test or ANOVA, and *P* < 0.05 was regarded as statistically significant. The data of statistical analysis was analyzed using SPSS 20.0 software and GraphPad Prism 8.

## Results

### TROP2 is aberrantly overexpressed in lung cancer and correlates with poor prognosis

Given the established role of TROP2 as a protein broadly overexpressed in many human cancers (Fig. S1), we sought to investigate its potential function in regulating a novel biological process—ferroptosis in the context of non-small cell lung cancer (NSCLC). We first analyzed TROP2 expression in human NSCLC tissues. Data from the GEPIA database revealed that *TROP2 (TACSTD2)* expression was markedly higher in both lung adenocarcinoma (LUAD) and lung squamous cell carcinoma (LUSC) than in normal tissues (Fig. 1A). Single-cell RNA sequencing (scRNA-seq) data further confirmed elevated *TROP2* levels in lung cancer tissues, with its expression predominating in epithelial cells (Fig. 1B-C). Clinically, high TROP2 expression was associated with poor prognosis in lung cancer patients (Fig. 1D). To validate these findings, we examined TROP2 protein expression via immunohistochemistry (IHC) in 69 paired tumor and adjacent normal tissues. Representative IHC images demonstrated a clear difference in TROP2 expression between lung cancer and adjacent normal tissues (Fig. 1E). Quantification of the IHC results revealed that 40% of lung cancer tissues exhibited high TROP2 expression, 44% showed moderate expression, and 16% had low expression (Fig. 1F). Furthermore, we assessed TROP2 mRNA levels in the normal lung cell line BEAS-2B and several lung cancer cell lines using quantitative RT-PCR (Fig. 1G). Western blot analysis confirmed higher TROP2 protein expression in H292, PC9 and ChaGO-K-1 cells compared to normal lung cells (Fig. 1H). These results suggest that TROP2 is upregulated in NSCLC and may contribute to tumor progression. The clinical correlation further suggests that TROP2 likely plays a role in lung cancer progress.

### TROP2 regulates the malignant phenotype of NSCLC cells

Given that TROP2 is upregulated in most lung cancer tissues, we hypothesized it might promote cancer cell proliferation and metastasis. To test this, we performed functional assays in lung cancer cell lines. Based on the TROP2 expression profiles (Fig. 1G, H), we selected PC9 and H292 cells for further study. TROP2 was effectively knocked down in these cells using RNA interference (RNAi), as confirmed by qRT-PCR and Western blotting (Fig. 2A, B). Subsequent functional assays revealed that TROP2 knockdown markedly impaired cell proliferation (Fig. 2C, D) and colony formation capacity (Fig. 2E). Furthermore, sphere formation assays indicated that TROP2 downregulation suppressed tumor spheroid growth (Fig. 2F). Together, these results suggest that TROP2 inhibition attenuates lung cancer cell survival ability in vitro. Consistent with these findings, shRNA-mediated TROP2 knockdown yielded similarly suppressed cell proliferation (Fig. S2). Conversely, TROP2 overexpression enhanced oncogenic properties. In PC9 and A549 cells with forced TROP2 expression, we observed increased proliferation (Fig. 2G, H), colony formation (Fig. 2I) and sphere-forming ability (Fig. 2J). Collectively, these data demonstrate that TROP2 plays a critical role in promoting NSCLC cell growth progression.

### TROP2 inhibition potentiates ferroptosis in lung cancer cells

For further exploring the roles of TROP2 in lung cancer, we preformed transcriptome sequencing on H292 cells following TROP2 knockdown. A heatmap displayed the top 24 genes differentially expressed genes in TROP2-deficient cells (Fig. 3A). KEGG pathway analysis revealed that *TROP2* is involved in ferroptosis regulation (Fig. 3B). We validated the expression of ferroptosis-associated genes by qRT-PCR and found that *HMOX1* was the most remarkably upregulated gene upon TROP2 suppression. Other key ferroptosis-related genes, such as *SLC7A11*, were downregulated, while *ACSL4* and *FTH1* showed a moderate increase (Fig. 3C). To further characterize *TROP2*-mediated ferroptosis, we assessed reactive oxygen species (ROS) levels by flow cytometry. *TROP2* knockdown led to a marked increase in ROS in both H292 and PC9 cells (Fig. 3D, E). Additionally, glutathione (GSH) levels decreased (Fig. 3F-G), while MDA levels increased (Fig. 3H, I). TEM revealed classic ferroptotic morphological changes, including diminished or absent mitochondrial cristae, mitochondrial shrinkage, and increased electron density (Fig. 3J). At the molecular level, ferroptosis-related proteins SLC7A11 and GPX4 were downregulated, whereas ACSL4 was upregulated in TROP2-deficient H292 and PC9 cells (Fig. 3K). These findings demonstrate that TROP2 inhibition induces ferroptosis in lung cancer cells, highlighting its potential role in modulating redox homeostasis and iron-dependent cell death.

### TROP2 inhibition enhances RSL3-induced ferroptosis in NSCLC cells

To investigate whether TROP2 modulates ferroptosis specifically, we first used pharmacological inhibitors targeting other major cell death pathways. The results showed that only the ferroptosis inhibitor Ferrostatin-1 (Fer-1), but not inhibitors of other pathways, could rescue the cell death induced by the combination of TROP2 knockdown and RSL3 in PC9 cells, confirming the ferroptosis-specific nature of this efect (Fig. 4A). In TROP2-knockdown H292 cells, RSL3 treatment resulted in a more pronounced suppression of cell viability compared to controls, as shown by CCK8 assay (Fig. 4B). LIVE/DEAD staining further confirmed that TROP2 knockdown sensitized cell to RSL3-induced death, an effect that was rescued by the ferroptosis inhibitor Ferrostatin-1 (Fer-1) (Fig. 4C). We next examined key ferroptosis-related biochemical changes. TROP2 knockdown combined with RSL3 treatment substantially elevated lipid peroxidation (LPO) levels (Fig. 4D, E). Consistently, MDA levels were increased (Fig. 4F, G), while GSH levels were decreased (Fig. 4H, I) in TROP2-deficient cells upon RSL3 exposure. Finally, TEM revealed more severe ferroptotic morphological alterations, such as mitochondrial shrinkage and increased membrane density in TROP2-knockdown cells treated with RSL3 (Fig. 4J). In another cell line, we observed the similar results (Fig. S3). In contrast, TROP2 overexpression conferred resistance to RSL3-induced ferroptosis (Fig. S3). Collectively, these findings demonstrate that loss of TROP2 enhances the sensitivity of NSCLC cells to RSL3-induced ferroptosis.

### TROP2 suppresses ferroptosis by transcriptionally repressing HMOX1 in NSCLC cells

Transcriptomic analysis of TROP2-knockdown cells identified the ferroptosis-related gene HMOX1 as a key downstream candidate, leading us to hypothesize that HMOX1 mediates the regulatory effect of TROP2 on ferroptosis. To validate this, we first confirmed that TROP2 knockdown notably upregulated HMOX1 expression at both the mRNA (Fig. 5A) and protein levels (Fig. 5B) in H292 and PC9 cells. TROP2 down-regulation led to a markedly upregulation of HMOX1 (Fig. 5C). Genetic rescue experiments were then employed to establish causality. The enhanced sensitivity to RSL3-induced cell death caused by TROP2 knockdown was effectively reversed by co-knockdown of HMOX1 (Fig. 5D, E). Correspondingly, HMOX1 inhibition abrogated the elevated levels of lipid peroxidation (Fig. 5F, G), the increased MDA accumulation (Fig. 5H, I) and the depletion of GSH (Fig. 5J, K) observed in cells with TROP2 down-regulation. Consistently, TROP2 knockdown altered the protein levels of key ferroptosis regulators (SLC7A11, GPX4, ACSL4) were altered by in an HMOX1-dependent significant negative correlation between TROP2 and HMOX1 expression in human lung cancer specimens (Fig. S4I, J). In summary, these data demonstrate that TROP2 functions as a transcriptional repressor of HMOX1, and that the HMOX1 downregulation constitutes a key mechanmanner (Fig. 5L). Reverse rescue experiments further confirmed that HMOX1 overexpression resensitized TROP2-overexpressing cells to RSL3 (Fig. S4A-D) and mitigated the corresponding changes in GSH (Fig. S4E, F) and MDA (Fig. S4G, H). The clinical relevance of this axis was supported by the ism through which TROP2 constrains ferroptosis in NSCLC cells.

### TROP2 confers ferroptosis resistance by inactivating YAP to suppress HMOX1 transcription

Our transcriptomic data revealed that TROP2 knockdown concurrently upregulates YAP1. Given the established role of nuclear YAP as a transcriptional co-activator, along with our prior identification of HMOX1 as a key ferroptosis mediator downstream of TROP2, we hypothesized that YAP activation might drive HMOX1 expression. To test this, we conducted loss-of-function experiments. We first confirmed that TROP2 knockdown increased, while its overexpression decreased YAP1 mRNA levels (Fig. 6A, B). Critically, TROP2 inhibition robustly promoted YAP nuclear translocation (Fig. 6C, E, F), whereas TROP2 overexpression retained YAP in the cytoplasm (Fig. 6D), indicating that TROP2 negatively regulates YAP activity. Inhibiting YAP in TROP2-knockdown cells attenuated ferroptosis (Fig. 6G, H, I, J, K, L). Indeed, YAP knockdown suppressed the upregulation of HMOX1 (Fig. S5A-C), placing YAP upstream of HMOX1 in this pathway. YAP robustly activated the HMOX1 promoter in a luciferase reporter assay. Truncation analysis mapped the essential responsive element to a region between − 500 and − 200 bp, as its deletion abolished activation (Fig.S5D-E). Rescue experiments further validated the physiological relevance of this axis: restoring YAP expression in HMOX1 knockdown cells re-sensitized them to resistance, evidenced by decreased viability (Fig. S5F, G), pro-ferroptotic shifts in GSH (Fig. S5H, I) and MDA (Fig. S5J, K) levels. In summary, building on a transcriptomic lead, we have identified a coherent signaling module: TROP2 maintains ferroptosis resistance by inactivating YAP, thereby repressing the expression of the critical ferroptosis mediator HMOX1.

### TROP2 suppression sensitizes NSCLC to RSL3-induced ferroptosis In Vivo

To evaluate the combined effect of TROP2 knockdown and RSL3 treatment in vivo, we established a xenograft mouse model using PC9 cells with stable TROP2 knockdown (shTROP2) or control (shCON). Mice were randomized into four groups (*n* = 6 per group) and treated with RSL3 (5 mg/kg) or vehicle via intraperitoneal injection every other day, following the schedule (Fig. 7A). Consistent with our in vitro findings, TROP2 knockdown alone markedly inhibited tumor growth, resulting in smaller and lighter tumors compared to the shCON group (Fig. 7B, D). RSL3 monotherapy also moderately suppressed tumor growth in control tumors. Notably, the combination of TROP2 knockdown and RSL3 treatment produced the most potent antitumor effect, with tumors exhibiting the smallest volumes and lowest weights among all groups (Fig. 7C, D). To confirm that the enhanced tumor suppression was mediated through ferroptosis activation, we examined key ferroptosis markers in tumor tissues. IHC analysis revealed that tumors from the shTROP2 + RSL3 group exhibited the highest expression of HMOX1 and ACSL4, along with the lowest expression of SLC7A11 (Fig. 7E). Furthermore, we directly measured lipid peroxidation levels in tumor lysates and found a marked increase in MDA content, along with a concomitant decrease in GSH, specifically in the combination treatment group (Supplementary Fig. S6A, B). These biochemical changes confirm the induction of a robust ferroptotic response in vivo. Collectively, our in vivo data demonstrate that TROP2 depletion not only attenuates tumor growth autonomously but also profoundly sensitizes NSCLC tumors to RSL3-induced ferroptosis, underscoring the therapeutic potential of targeting the TROP2 pathway in combination with ferroptosis inducers.

## Discussion

The diagnosis and treatment of lung cancer have improved continuously with the advancement of medical technology. However, its incidence remains high due to metastasis and therapeutic resistance, underscoring the need for novel therapeutic strategies. In this study, we investigated the expression and functional role of TROP2 in lung cancer progression. We found that inhibiting TROP2 sensitizes lung cancer cells to ferroptosis and suppressed cell proliferation. This effect was achieved by modulating YAP nuclear translocation, which activated the downstream ferroptosis-related factor HMOX1 (Fig. 8).

Ferroptosis is a complex biological process of iron-dependent cell death driven by lipid peroxidation and plays a crucial role in tumor microenvironment [2]. Its susceptibility varies across different cancer cell types, influenced by their distinct metabolism states and epigenic backgrounds [3]. TROP2 is known to play critical roles in cancer cellular proliferation, metastasis, angiogenesis, immune modulation, drug resistance and poor prognosis [7, 8]. Given its pivotal functions, TROP2 has emerged as a successful target for antibody-drug conjugates (ADCs) in clinical use currently, such as sacituzumab govitecan [17]. Mechanistically, TROP2 regulates diverse cancer cell biological processes through multiple signaling pathways, including protein kinase C (PKC), MAPK, JAK/STAT3 and PI3K/AKT [7, 8]. Beyond these established roles, our study identifies TROP2 is a key regulator of ferroptosis in NSCLC for the first time. We found that knockdown of TROP2 effectively suppressed cell proliferation and migration while markedly enhancing sensitivity to the ferroptosis inducer RSL3. This increased sensitivity was evidenced by increased lipid peroxidation and reactive oxygen species (ROS) accumulation.

Heme oxygenase 1 (HMOX1, also known as HO1) is a key enzyme in heme metabolism, responsible for converting heme into biliverdin [18]. Specifically, HMOX1 catalyzes the degradation of heme into biliverdin, carbon monoxide and iron. HMOX1 plays a dual role in driving and regulating ferroptosis. The catalytic effect of HMOX1 can lead to the accumulation of iron, thereby driving the process of iron death in cells. The role of HMOX1 in ferroptosis is context-dependent and paradoxical [19–22]. It can exert pro-ferroptotic effects by promoting intracellular iron accumulation and subsequent lipid peroxidation [8, 9]. Conversely, its degradation products, biliverdin/bilirubin and CO, possess antioxidant and anti-inflammatory properties, which may confer anti-ferroptotic resistance [10, 23]. This functional duality likely depends on the cellular microenvironment, iron-handling capacity and disease state. In our NSCLC model, we consistently observed that TROP2 inhibition led to HMOX1 upregulation, which was associated with increased ferroptotic markers (e.g., MDA, ROS) and decreased cell viability upon RSL3 treatment. Furthermore, HMOX1 knockdown rescued the ferroptosis sensitivity induced by TROP2 deficiency. These findings strongly suggest that, within the specific context of our study, the net effect of TROP2-regulated HMOX1 expression is pro-ferroptotic, likely by tipping the balance toward lethal iron overload and oxidative stress.

Emerging evidence links the Hippo signaling pathway to ferroptosis regulation. The core effector of this pathway, YAP/TAZ, upon Hippo pathway inactivation, translocates to the nucleus to drive the transcription of target genes involved in growth and survival. Previous studies showed that YAP/TAZ activation promotes its cytoplasmic localization, proteasome degradation and then reduces transcriptional activity [24–30]. YAP promotes ferroptosis by upregulating several ferroptosis modulators, including ACSL4, transferrin receptor 1 (TFRC) or by downregulating anti-ferroptotic genes such as SLC7A11 in certain contexts. SUFU could suppress breast cancer ferroptosis by activating Hippo/YAP [25]. YAP could be activated by LACAT3 [26], CYLD [27], ZDHHC7 [28] and LATS2 [29]. Wu et al. have found that E-cadherin suppressed ferroptosis by activating NF2 and Hippo signaling pathway in epithelial cells [26]. Also, another study has found that YAP/TAZ activation made cancer cells sensitive to ferroptosis but resistant to apoptosis [27]. These studies highlight targeting the Hippo/YAP signaling might provide therapeutic potential for ferroptosis-inducing cancer therapy. However, the mechanisms underlying the regulation of YAP/TAZ signaling in ferroptosis remain unclear. In our study, we found that inhibiting TROP2 could inhibited the activation of Hippo signal pathway by upregulating HMOX1. Therefore, targeting ferroptosis may have therapeutic efficacy in lung cancer with YAP activation. This indicates that YAP, through its regulatory role, affects the balance between cell survival and death, especially playing a key role in combating drug resistance in diseases such as cancer.

Our data establish that TROP2 regulates ferroptosis primarily through the YAP-HMOX1 axis. To systematically address its broader impact on the ferroptosis network, we analyzed transcriptomic data from TROP2-knockdown cells. The heatmap revealed specific upregulation of pro-ferroptotic genes (e.g., HMOX1) alongside downregulation of key defense genes (e.g., SLC7A11) (Fig. S8A), consistent with our phenotypic findings. Importantly, to investigate potential crosstalk with the major antioxidant regulator *NFE2L2*, we performed RT-PCR analysis, which showed that TROP2 knockdown did not alter *NFE2L2* levels (Fig. S8B). These results together indicate that the ferroptosis sensitization induced by TROP2 inhibition is mediated through a specific transcriptional reprogramming largely independent of the NRF2/KEAP1 pathway, further underscoring the uniqueness of the TROP2-YAP-HMOX1 regulatory axis.

To begin exploring the translational relevance of the TROP2-YAP-HMOX1 axis across different genomic contexts, a point raised during review, we analyzed its correlation in the Cancer Genome Atlas Lung Adenocarcinoma (TCGA-LUAD) cohort. Notably, TROP2 expression was significantly elevated in tumors harboring EGFR mutations compared to EGFR wild-type tumors (Fig. S9A). In contrast, no significant association was observed with TP53 mutation status (Fig. S9B). This bioinformatic evidence suggests that the pathogenic role of TROP2, and potentially its associated ferroptosis vulnerability, may be particularly pronounced in the EGFR-mutant LUAD subtype, a population of high clinical importance and often associated with targeted therapy resistance.

In conclusion, we have identified a novel TROP2-YAP-HMOX1 regulatory axis that governs ferroptosis resistance in NSCLC. This discovery not only expands the functional repertoire of TROP2 in cancer biology but also provides a mechanistic rationale for combining TROP2-targeted therapies (e.g., ADCs) with ferroptosis inducers. Such a combinatorial approach could potentially overcome therapeutic resistance and improve outcomes for NSCLC patients, positioning TROP2 as a promising molecular target for future therapeutic development.

## Supplementary Information

Below is the link to the electronic supplementary material.

Supplementary Material 1

Supplementary Material 2

Supplementary Material 3

Supplementary Material 4

Supplementary Material 5

Supplementary Material 6

Supplementary Material 7

Supplementary Material 8

Supplementary Material 9

Supplementary Material 10

Supplementary Material 11

Supplementary Material 12

## Data Availability

Any additional data and materials are available from corresponding authors on reasonable request.
