# Supplementary figures and images for: TROP2 confers resistance to oxidative stress-induced cancer cell death through YAP/HMOX1 signaling
Source: J Transl Med. 2026 Mar 11;24:438. doi: 10.1186/s12967-026-07955-z (PMC13034606; doi:10.1186/s12967-026-07955-z)

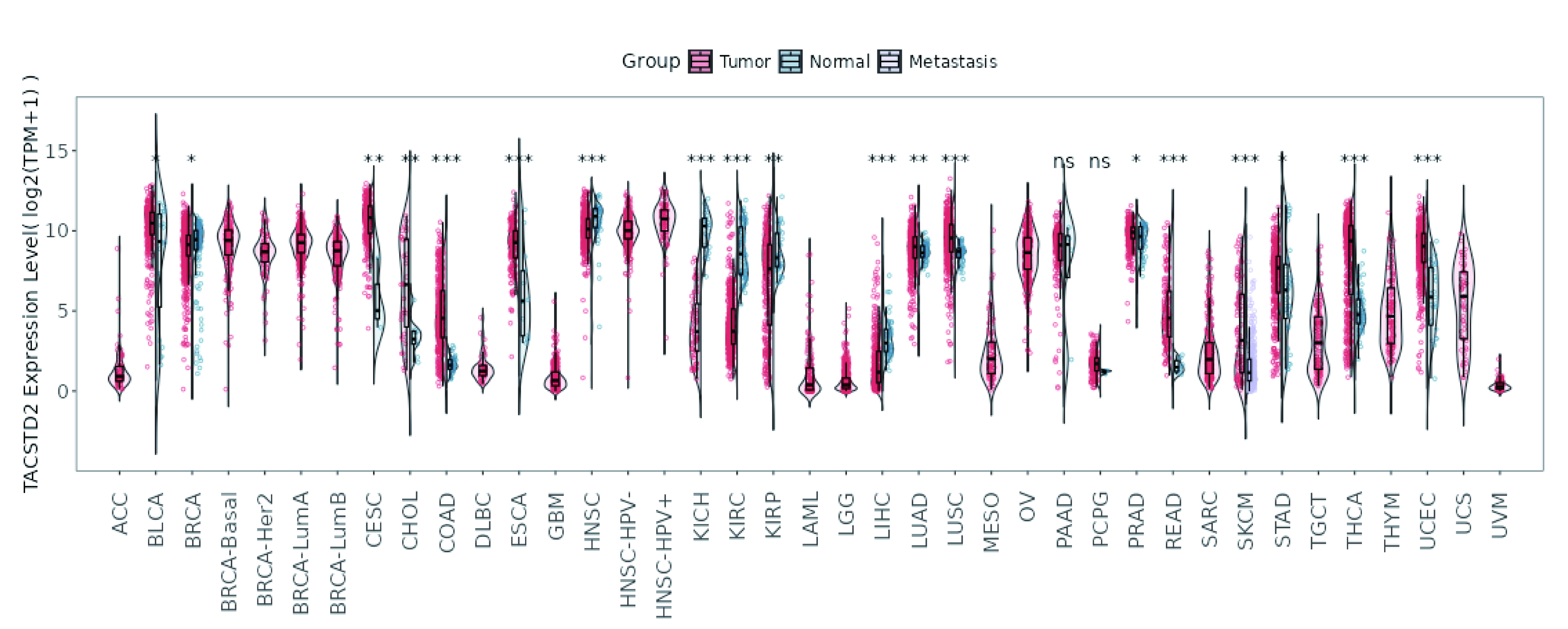

Supplement: Supplementary file 1 — Supplementary Material 1 [file 12967_2026_7955_MOESM1_ESM.tif]

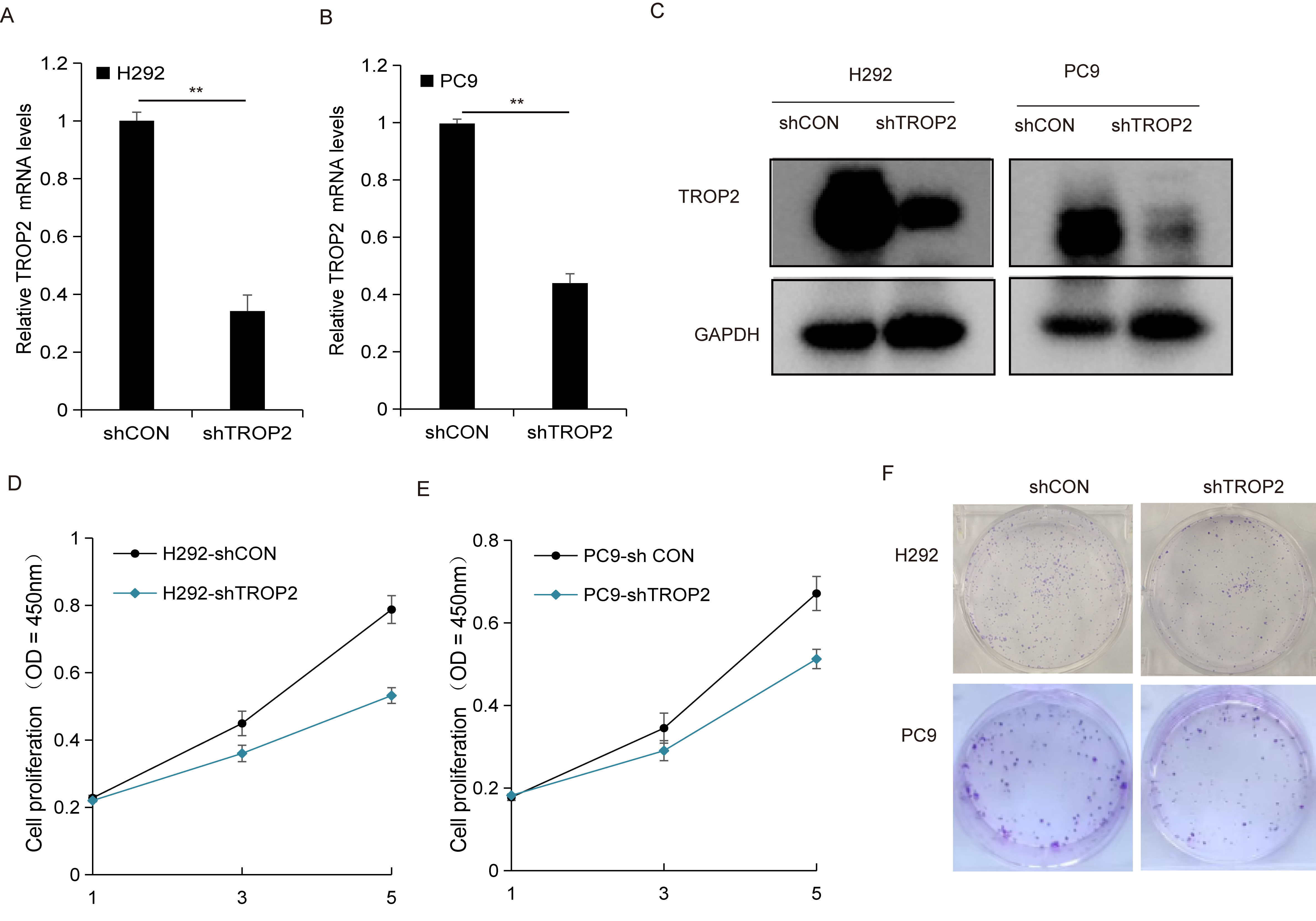

Supplement: Supplementary file 2 — Supplementary Material 2 [file 12967_2026_7955_MOESM2_ESM.tif]

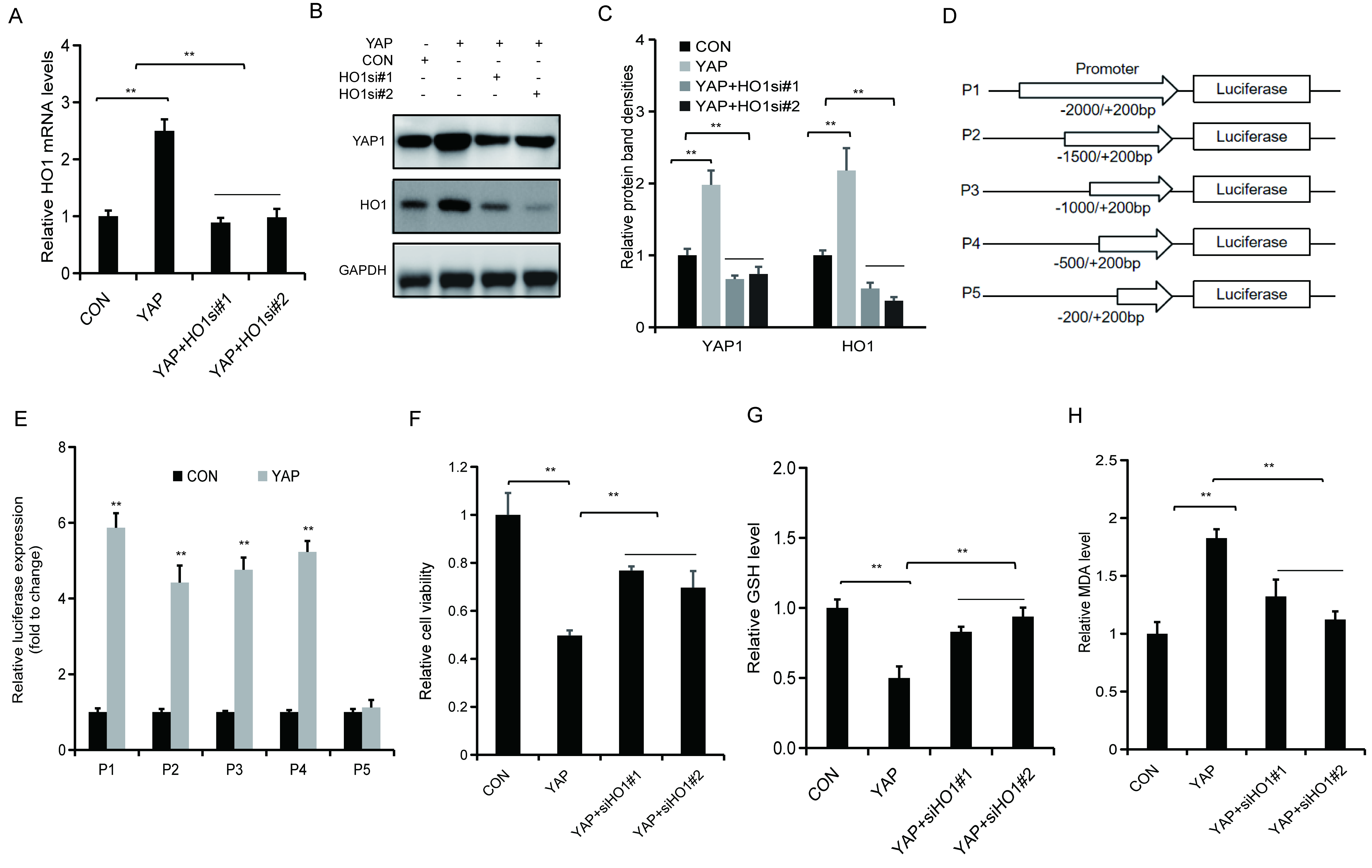

Supplement: Supplementary file 5 — Supplementary Material 5 [file 12967_2026_7955_MOESM5_ESM.tif]

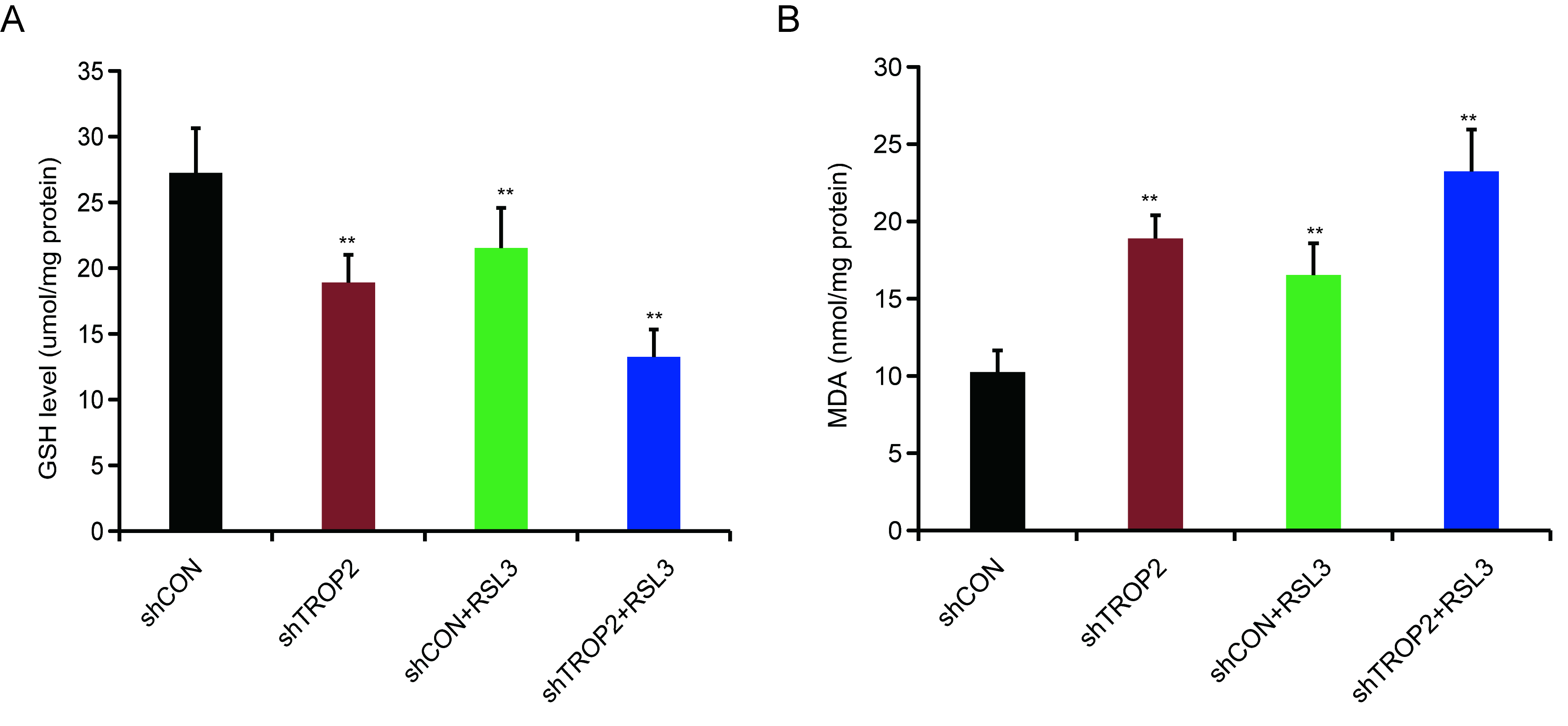

Supplement: Supplementary file 6 — Supplementary Material 6 [file 12967_2026_7955_MOESM6_ESM.tif]

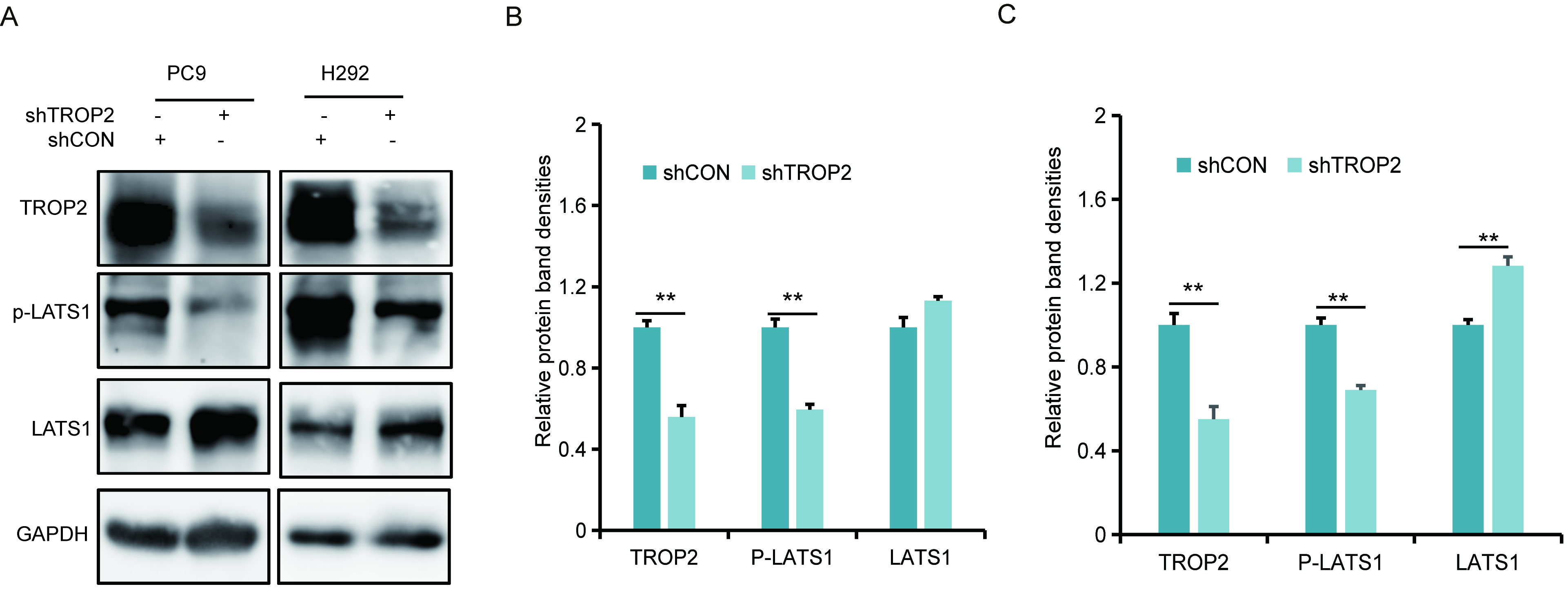

Supplement: Supplementary file 7 — Supplementary Material 7 [file 12967_2026_7955_MOESM7_ESM.tif]

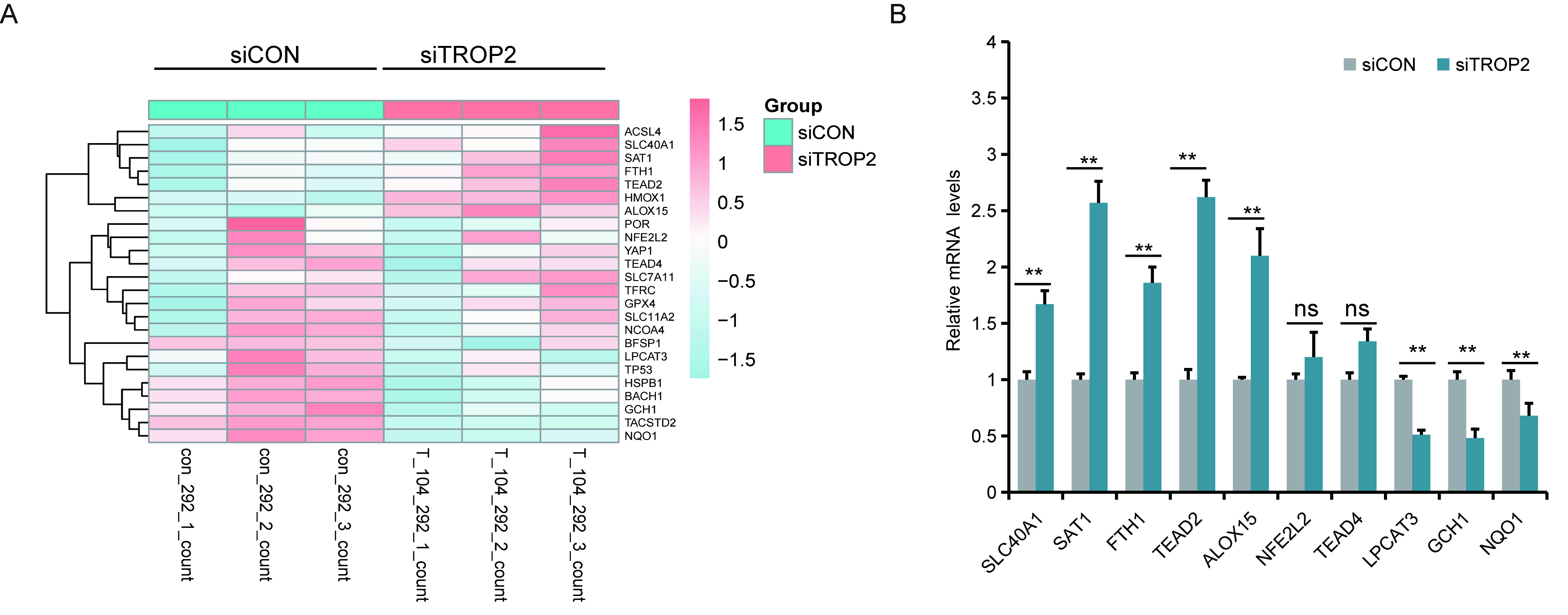

Supplement: Supplementary file 8 — Supplementary Material 8 [file 12967_2026_7955_MOESM8_ESM.tif]

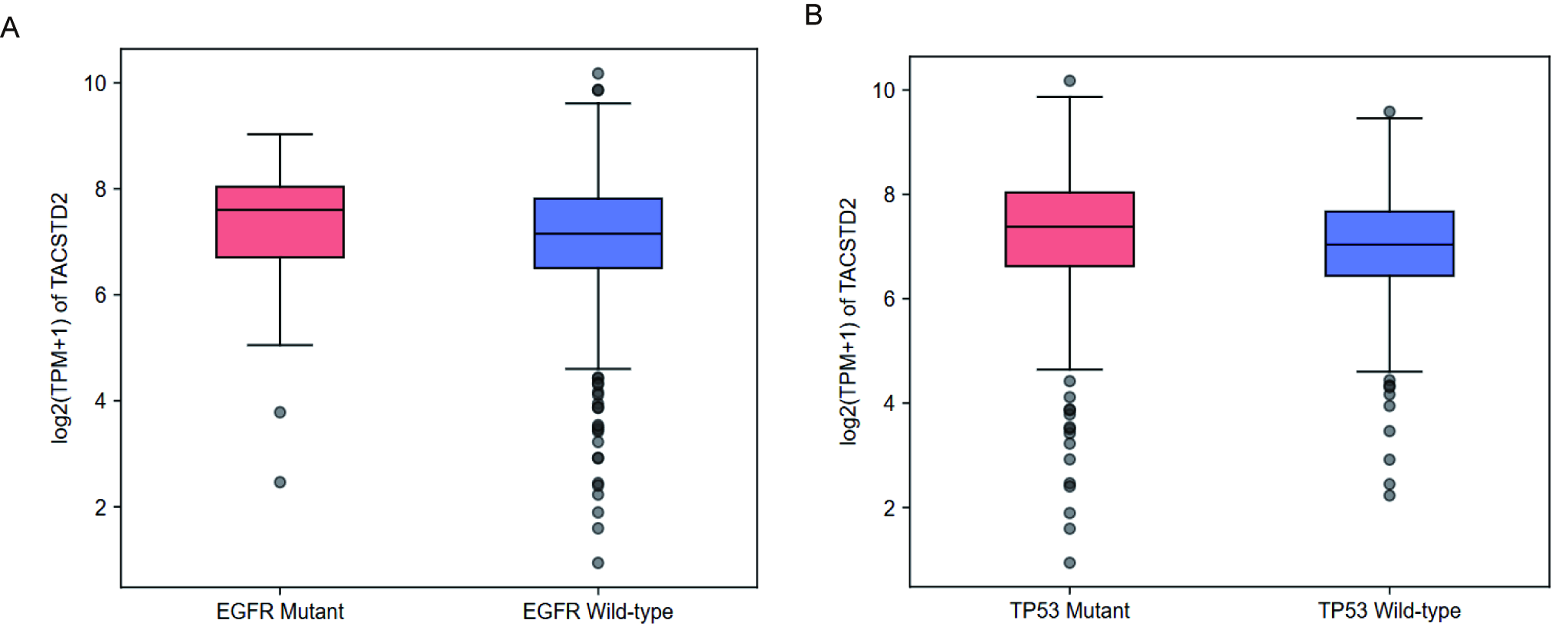

Supplement: Supplementary file 9 — Supplementary Material 9 [file 12967_2026_7955_MOESM9_ESM.tif]
