# Supplementary material for: TROP2 confers resistance to oxidative stress-induced cancer cell death through YAP/HMOX1 signaling
Source: J Transl Med. 2026 Mar 11;24:438. doi: 10.1186/s12967-026-07955-z (PMC13034606; doi:10.1186/s12967-026-07955-z)
Supplement: Supplementary file 10 — Supplementary Material 10 [file 12967_2026_7955_MOESM10_ESM.docx]

**Supplementary figure legends**

**Fig S1. TROP2 expressionlung tissues.** TROP2 mRNA levels in lung cancer samples (Timer).

**Fig S2. TROP2 promotes NSCLC cell growth.** TROP2 was down-regulated in PC9 and H292 cells with TROP2 shRNA lentivirus infection. TROP2 mRNA and protein levels were evaluated by real time RT-PCR (A-B) and Western blotting (C). (D-E) The cell viability was suppressed in PC9 and H292 cells with TROP2 knockdown. Cell proliferation was evaluated by CCK8. (F) The colonies were reduced in PC9 and H292 cells with TROP2 knockdown using colony formation assay. Data are shown as the mean ± SD (n = 3). Statistical significance was determined by two-tailed unpaired Student’s t-test for comparisons between two groups (A, B, D, E, F). *P < 0.05, **P < 0.01.

**Fig S3.** **TROP2 modulates ferroptosis sensitivity in NSCLC cells, related to Figure 4.** (A) Morphological assessment of ferroptosis in H292 cells. Representative bright-field images of H292 cells with or without TROP2 knockdown, treated with 5 µM RSL3 for the indicated time. TROP2 knockdown sensitized cells to RSL3, leading to the appearance of characteristic ferroptotic morphology, including cell rounding, membrane blebbing, and ballooning (enlarged, optically clear cytoplasm). (B) Protein levels in ChaGo-K-1 cells with or without TROP2 knockdown by western blotting assay. Quantitative analysis (bar graph) of the bands was shown adjacent to the representative blots. (C) The cell viability of ChaGo-K-1 cells with or without TROP2 knockdown, treated with RSL3, as determined using the CCK-8 assay. (D) GSH levels in ChaGo-K-1 cells were measured in TROP2 knockdown cells with or without 5uM RSL3 for 24h. (E-F) The relative cell viability of PC9 and A549 cells overexpressing TROP2 were treated by indicated dosages of RSL3 for 48 h. (G) Cell and death assay in PC9 and A549 cells. (H-I) The cellular GSH levels were assayed in TROP2 knockdown cells with or without 5uM RSL3 for 24h. (J-K) The cellular MDA levels were assayed in TROP2 knockdown cells with or without 10uM RSL3 for 24h. Data are shown as the mean ± SD (n = 3). For panels with multiple comparisons (C, D, E, F), statistical significance was determined by two-way ANOVA followed by Sidak’s post hoc test. For panels comparing two groups (G, H, I, J, K), a two-tailed unpaired Student’s t-test was used. *P < 0.05, **P < 0.01.

**Fig S4. HMOX1 is involved in TROP2 suppression mediated ferroptosis.** (A) HMOX1 mRNA levels in NSCLC cells with TROP2 overexpression. The gene expression verification in PC9 and A549 cells with TROP2 overexpression. HMOX1 mRNA was examined by real time RT-PCR. (B) HMOX1 protein in PC9 and A549 cells with TROP2 overexpression. HMOX1 protein levels were examined by western blotting. (C-D) PC9 and A549 cells with TROP2 overexpression infected with lentivirus mediated HMOX1, the cell viability of Cell growth ability was determined using the CCK-8 assay. (E-F) The cellular MDA levels were assayed. (G-H) The cellular GSH levels were assayed. (I) TROP2 and HMOX1 levels were in clinical lung cancer samples. (J) TROP2 was negatively related to HMOX1 in human lung cancer tissues (r=-0.501). HO1: HMOX1. Data are shown as the mean ± SD (n = 3). Statistical analysis: Two-tailed unpaired Student’s t-test (A, B); Two-way ANOVA followed by Sidak’s post hoc test (C-H); Pearson correlation analysis (J). *P < 0.05, **P < 0.01.

**Fig S5. YAP regulates HMOX1 induced ferroptosis. (A) mRNA levels of HMOX1 in PC9 cells. mRNA was evaluated by RT-PCR. (B)Protein levels were examined by Western blotting. (C)** Quantitative analysis (bar graph) of the bands was shown adjacent to the representative blots (B). (D-E) Luciferase activity of serial 5’-deletion constructs of the HMOX1 promoter in the presence of YAP overexpression, presented as fold activation relative to the empty vector control. The schematic above the graph illustrates the cloned promoter fragments. (F) Cell viability assessed by CCK-8 assay in PC9 cells with YAP overexpression, with or without concomitant HMOX1 downregulation. **(G)** Measurement of cellular GSH levels in the indicated groups of PC9 cells. **(H)**Measurement of cellular MDA levels in the indicated groups of PC9 cells.Data are shown as the mean ± SD (n = 3). Statistical analysis: Two-tailed unpaired Student’s t-test (A, B, C); One-way ANOVA followed by Tukey’s post hoc test (D, E); Two-way ANOVA followed by Sidak’s post hoc test (F, G, H). *P < 0.05, **P < 0.01.

**Fig S6. MDA and GSH levels in vivo.** (A) The cellular GSH levels were assayed in the tissues from animal models. (B) The cellular MDA levels were assayed in the tissues from animal models. Data are shown as the mean ± SD (n = 3). Statistical significance was determined by one-way ANOVA followed by Tukey’s post hoc test. *P < 0.05, **P < 0.01.

**Fig S7. TROP2 regulates the Hippo pathway upstream kinase LATS1.** (A) Western blot analysis of LATS1 and phosphorylated LATS1 (p-LATS1, Ser909) protein levels in control and TROP2-knockdown PC9 and H292 cells. GAPDH was used as a loading control. (B-C) Quantification of the band intensity for p-LATS1 and total LATS1 from (A), normalized to GAPDH. Data are shown as the mean ± SD (n = 3 independent experiments). Student’s t test was performed for statistical analysis. *P < 0.05, **P < 0.0.

**Fig S8. Analysis of ferroptosis-related gene expression upon *TROP2* knockdown in H292 cells.** (A) Heatmap depicting the expression changes of a curated set of ferroptosis-related genes in H292 cells with *TROP2* knockdown (sTROP2) compared to control (siCON), based on RNA sequencing data. Genes were selected for their established roles in ferroptosis. Each row represents a gene, each column a biological replicate. Expression levels are shown as Z-score (or log2 fold change). (B) Quantitative RT-PCR (qRT-PCR) validation of selected differentially expressed genes (including *SLC40A1, SAT1, FTH1, TEAD2, ALOX5, NFE2L2, TEAD4, LPCAT3, GCH1 and NQO1*). Expression levels are normalized to GAPDH and presented as fold change relative to siCON. Data are mean ± SD (n=3 independent experiments). Statistical significance was determined by two-tailed unpaired Student’s t-test. *P < 0.05, **P < 0.01.

**Fig S9. Association of TROP2 expression with major driver mutations in lung adenocarcinoma (LUAD).** (A) *TROP2* mRNA expression levels in the TCGA-LUAD cohort, stratified by EGFR mutation status (mutant vs. wild-type). (B) *TROP2* mRNA expression levels in the TCGA-LUAD cohort, stratified by TP53 mutation status (mutant vs. wild-type). Data are presented as box plots showing the median, interquartile range, and min-max values. The number of patients (n) in each group is indicated. Statistical significance was determined by the Mann-Whitney U test (or Student’s t-test if data are normally distributed). ns, not significant; *P < 0.05, **P < 0.01. Data source: The Cancer Genome Atlas Lung Adenocarcinoma (TCGA-LUAD) dataset.
