# Supplementary material for: TROP2 confers resistance to oxidative stress-induced cancer cell death through YAP/HMOX1 signaling
Source: J Transl Med. 2026 Mar 11;24:438. doi: 10.1186/s12967-026-07955-z (PMC13034606; doi:10.1186/s12967-026-07955-z)
Supplement: Supplementary file 12 — Supplementary Material 12 [file 12967_2026_7955_MOESM12_ESM.pptx]

## Slide 1
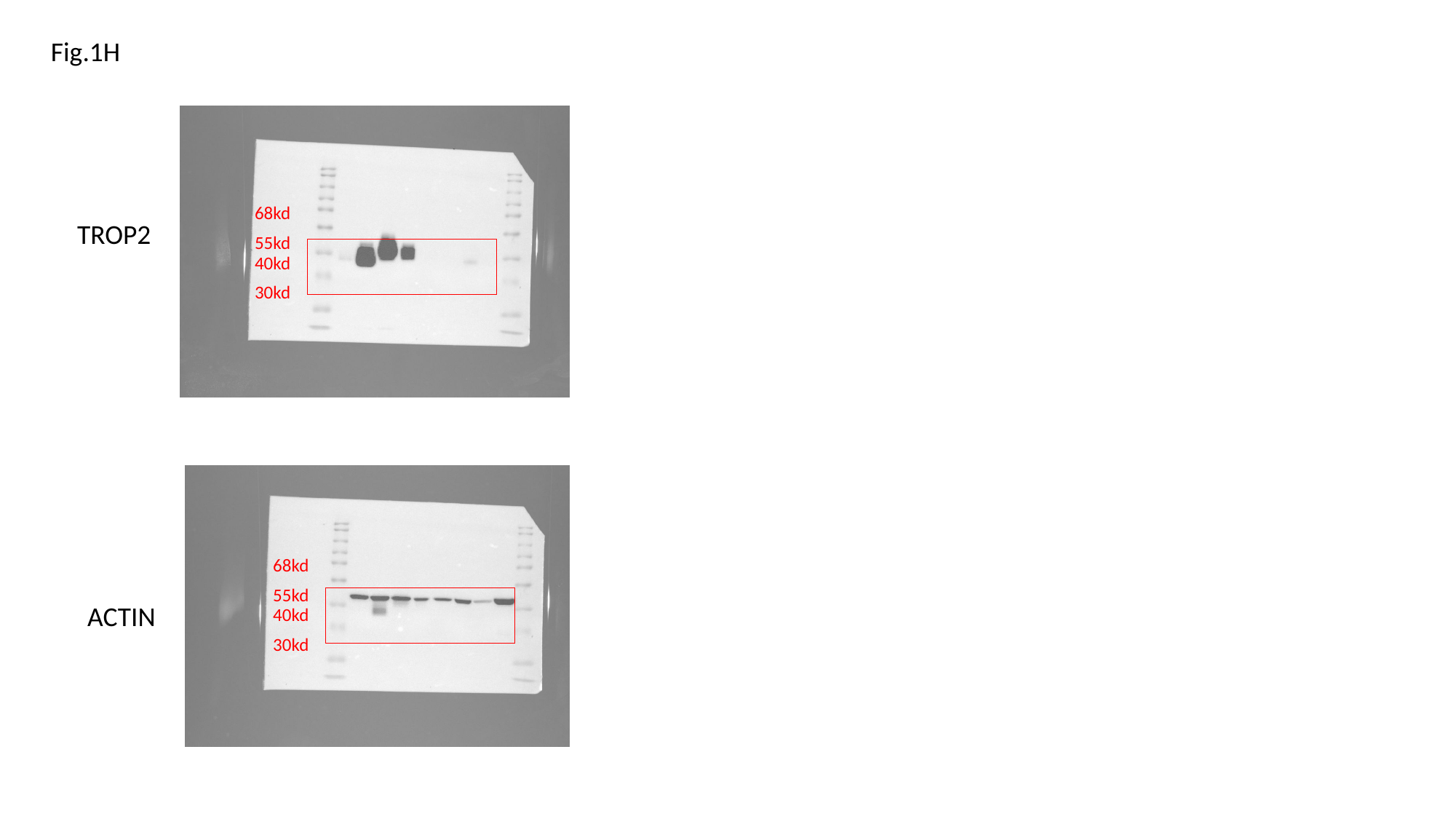

Fig.1H
68kd
TROP2
55kd
40kd
30kd
68kd
55kd
ACTIN
40kd
30kd

## Slide 2
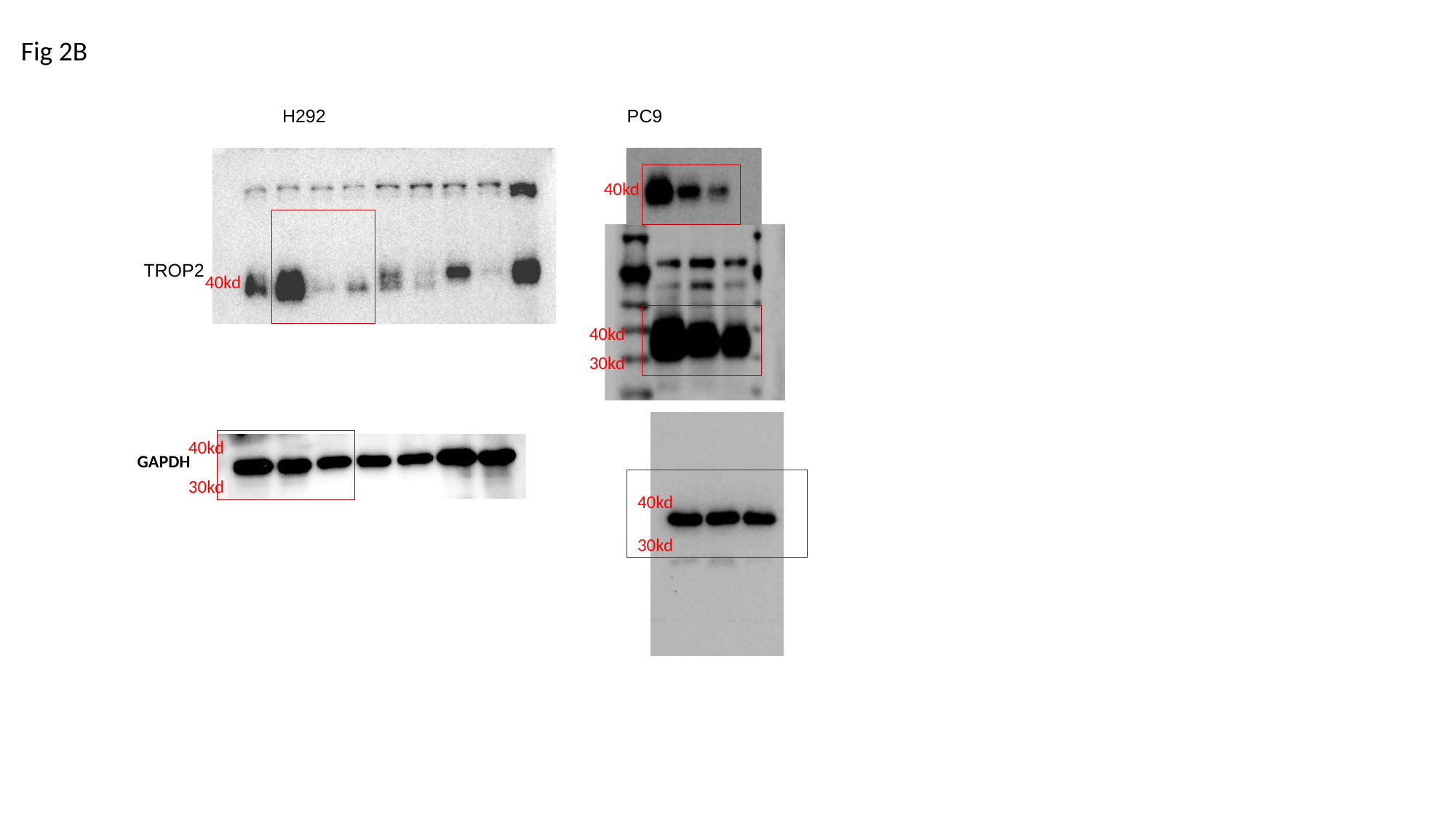

Fig 2B
H292
PC9
40kd
TROP2
40kd
40kd
30kd
40kd
GAPDH
30kd
40kd
30kd

## Slide 3
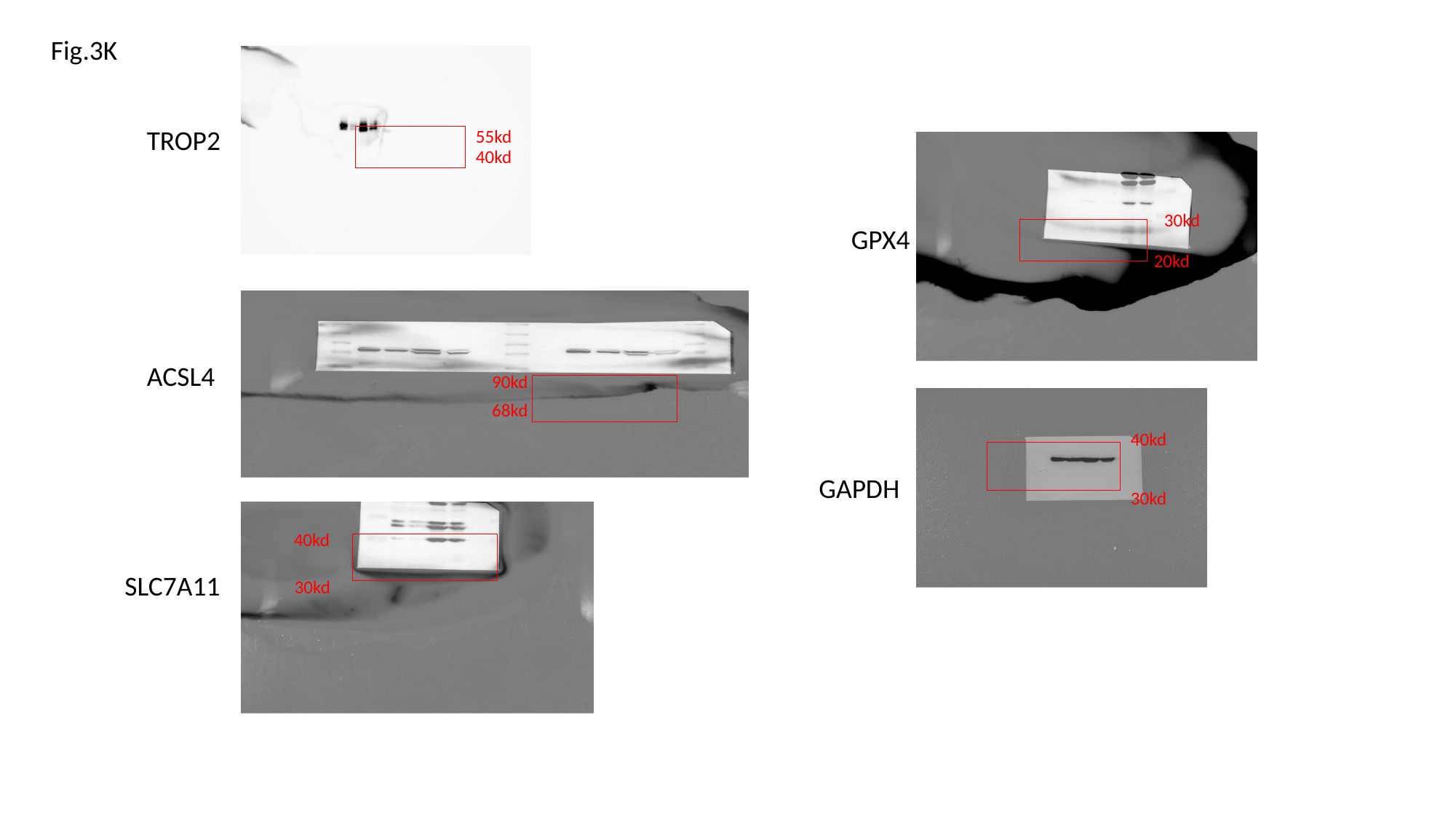

Fig.3K
TROP2
55kd
40kd
30kd
GPX4
20kd
ACSL4
90kd
68kd
40kd
GAPDH
30kd
40kd
SLC7A11
30kd

## Slide 4
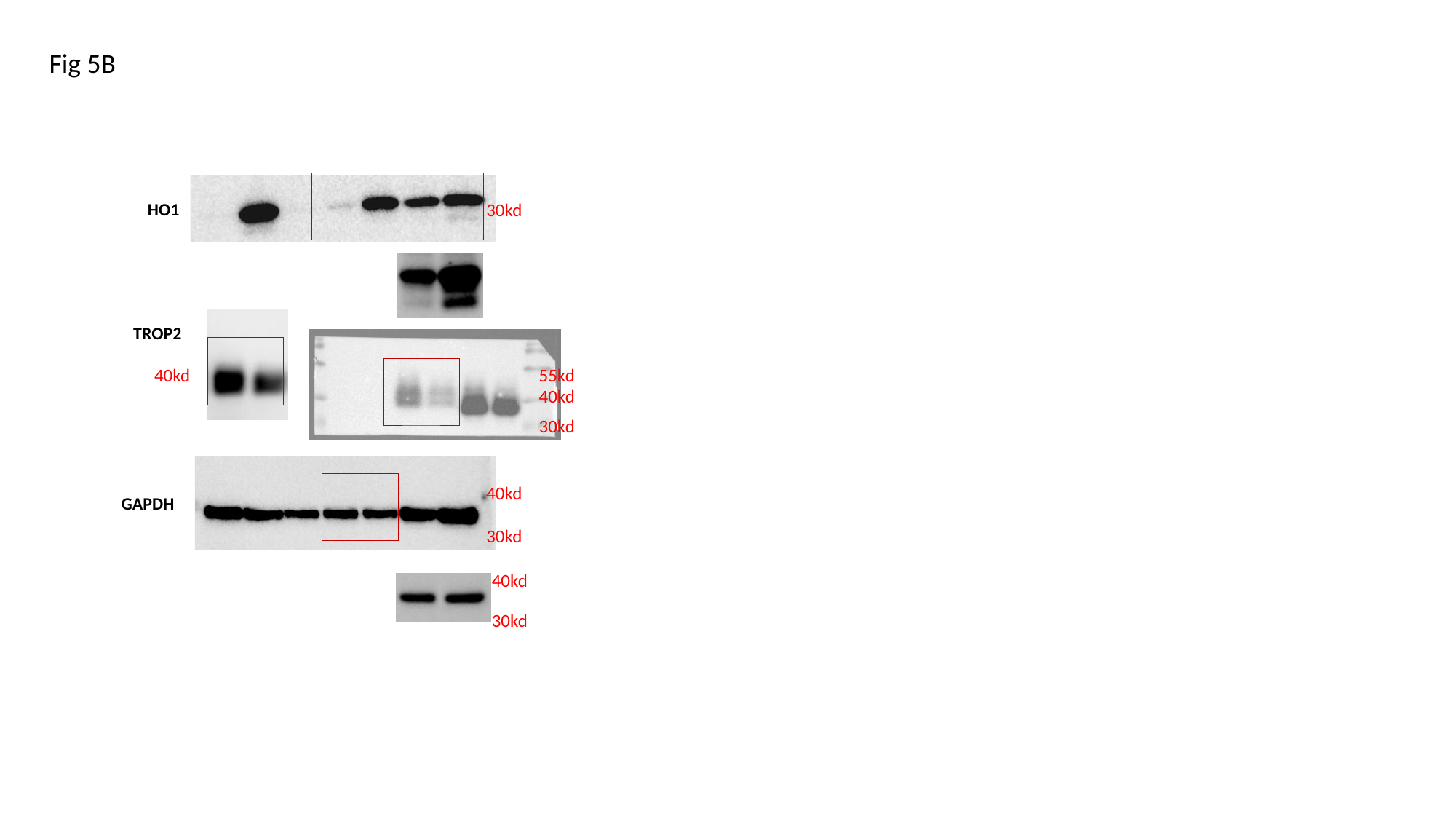

Fig 5B
HO1
30kd
TROP2
40kd
55kd
40kd
30kd
40kd
GAPDH
30kd
40kd
30kd

## Slide 5
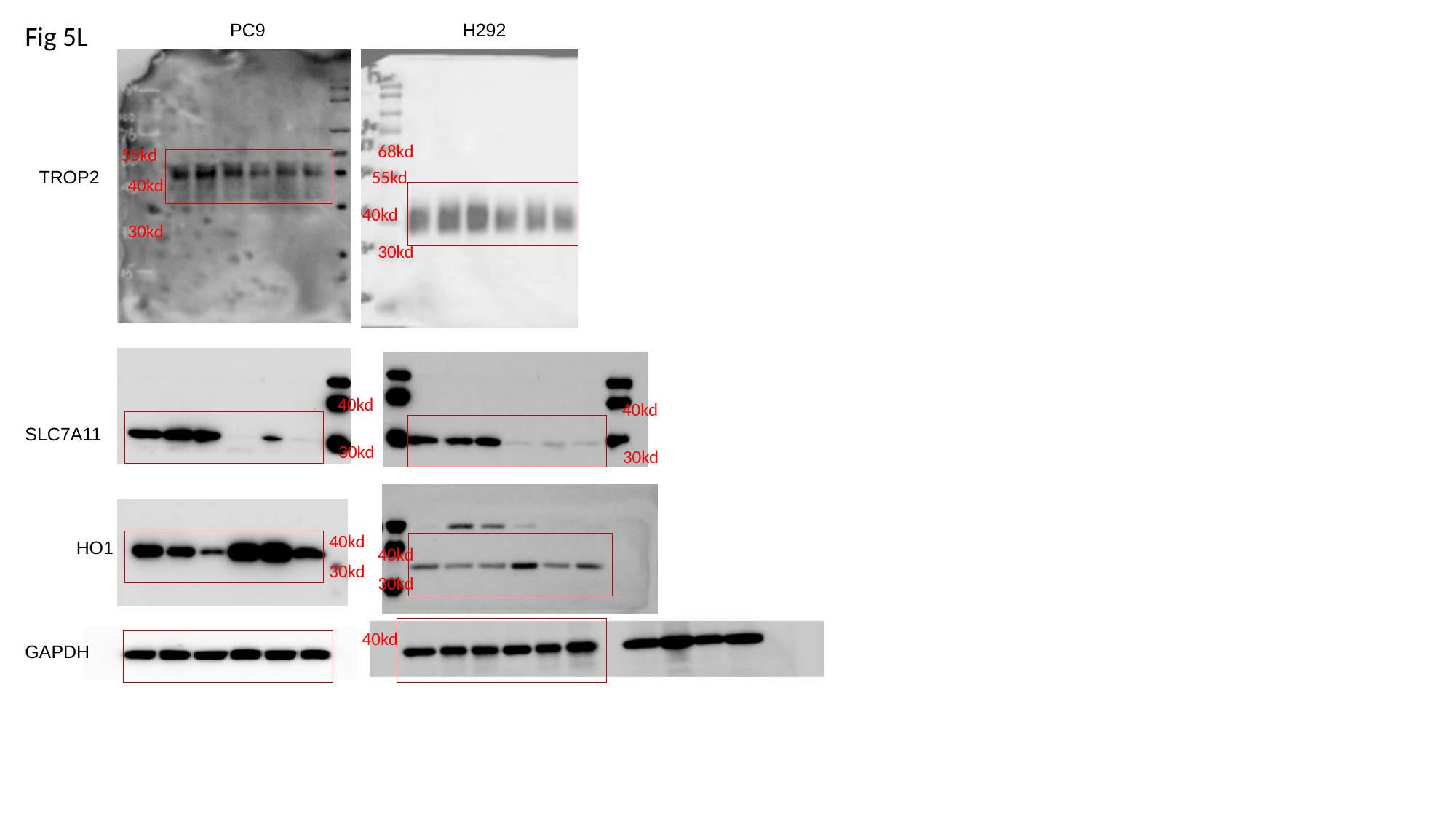

Fig 5L
PC9
H292
68kd
55kd
TROP2
55kd
40kd
40kd
30kd
30kd
40kd
40kd
SLC7A11
30kd
30kd
40kd
HO1
40kd
30kd
30kd
40kd
GAPDH

## Slide 6
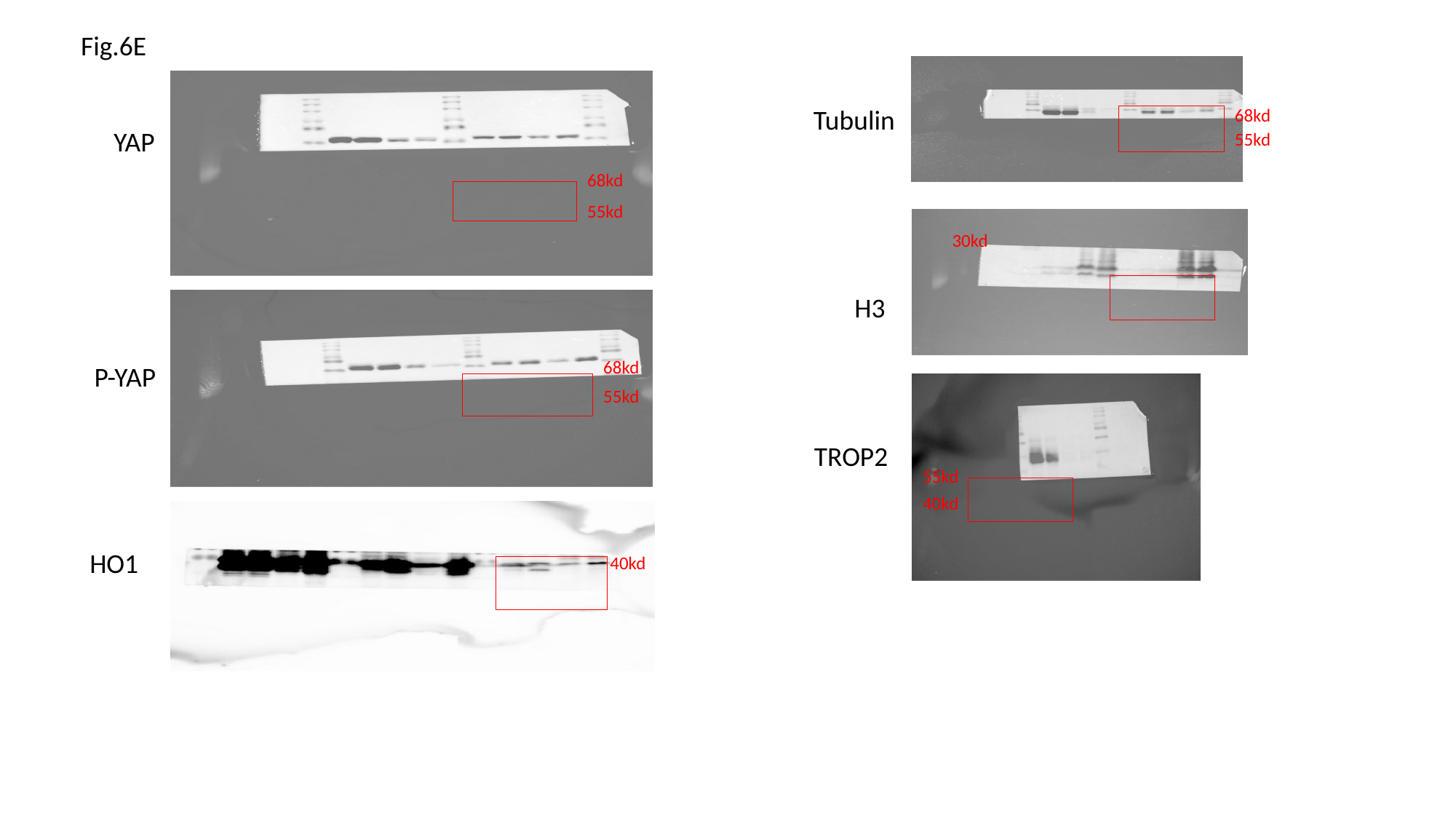

Fig.6E
Tubulin
68kd
YAP
55kd
68kd
55kd
30kd
H3
68kd
P-YAP
55kd
TROP2
55kd
40kd
HO1
40kd

## Slide 7
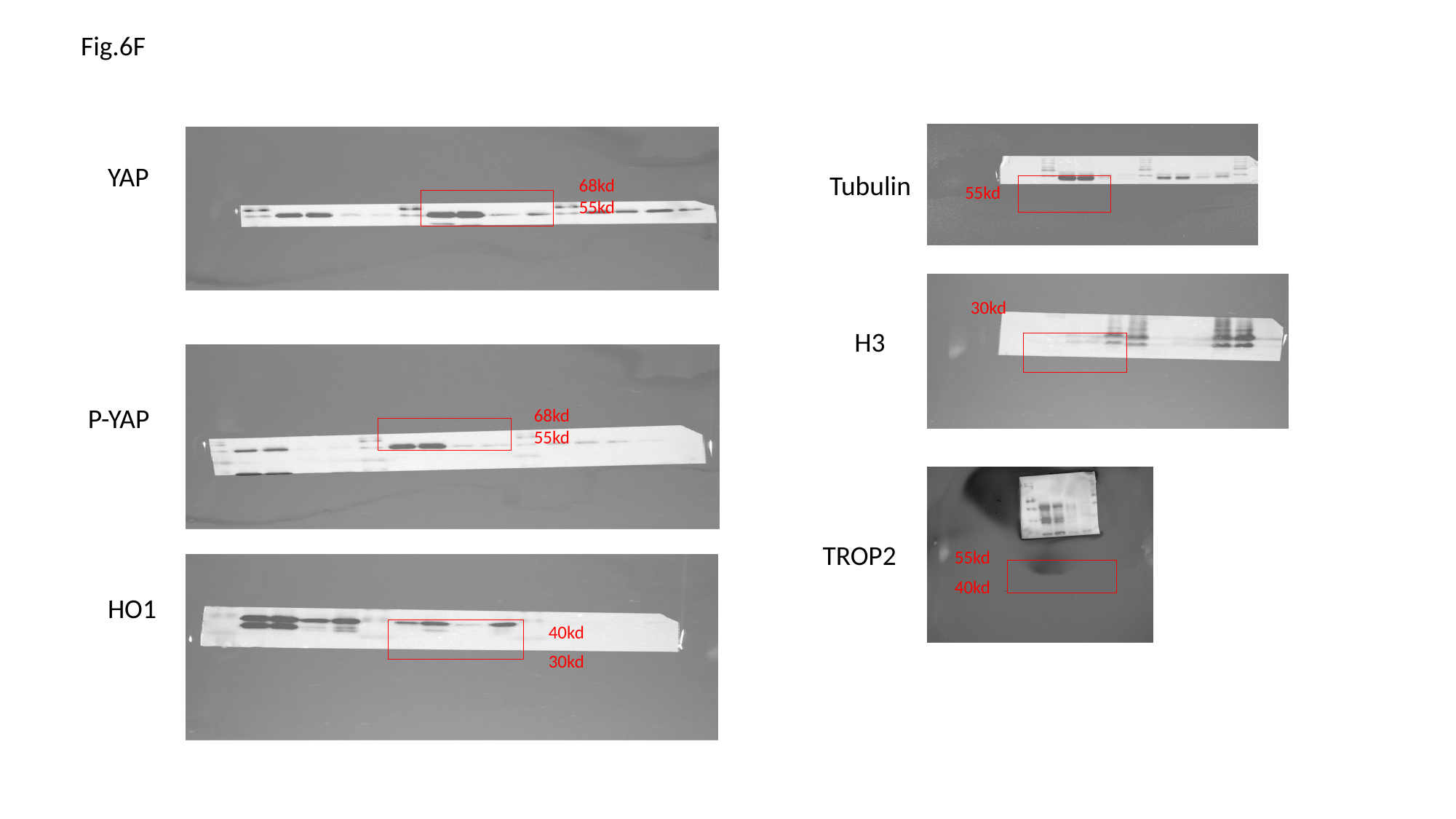

Fig.6F
YAP
Tubulin
68kd
55kd
55kd
30kd
H3
P-YAP
68kd
55kd
TROP2
55kd
40kd
HO1
40kd
30kd

## Slide 8
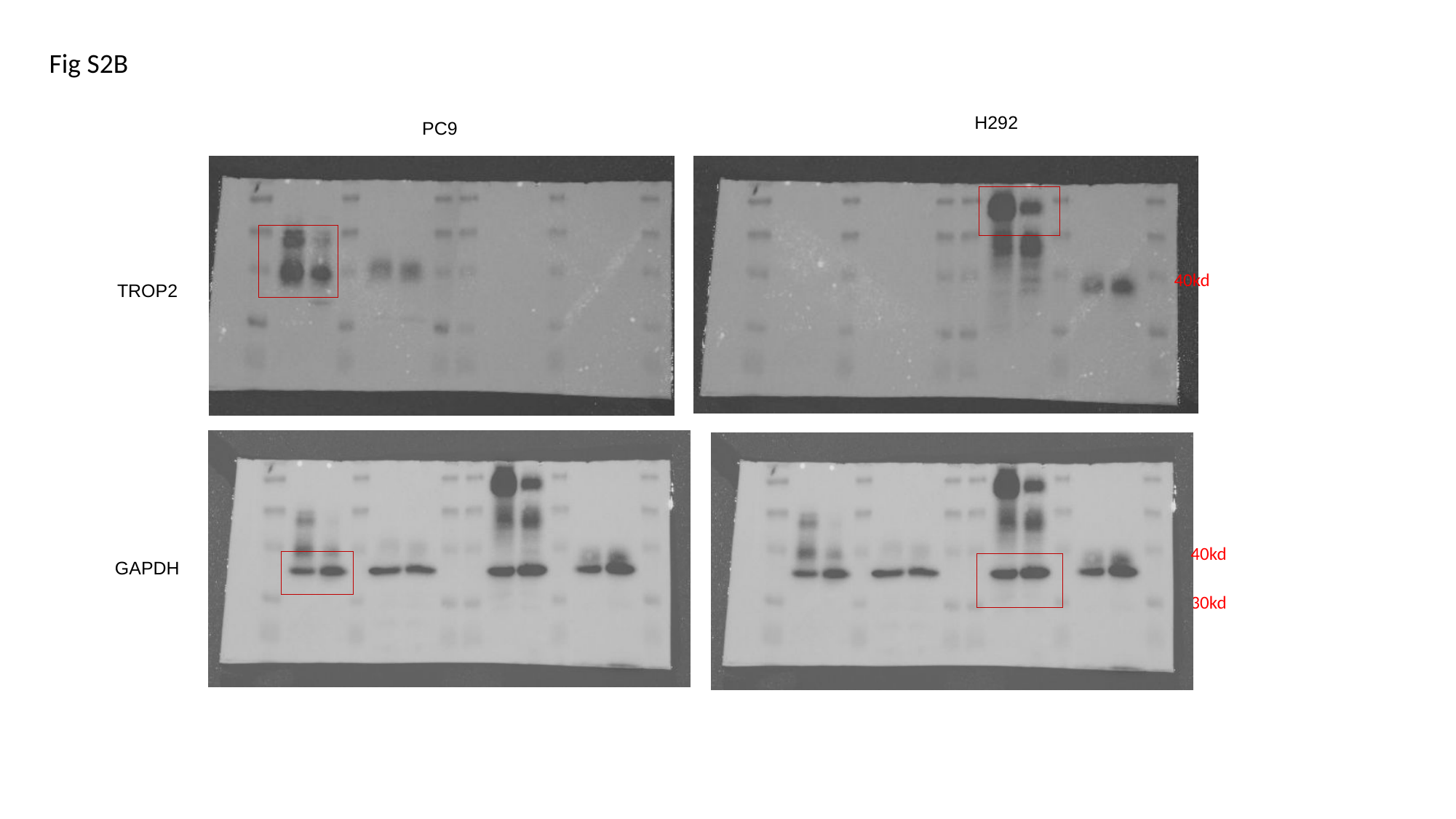

Fig S2B
H292
PC9
GAPDH
40kd
40kd
TROP2
40kd
GAPDH
30kd

## Slide 9
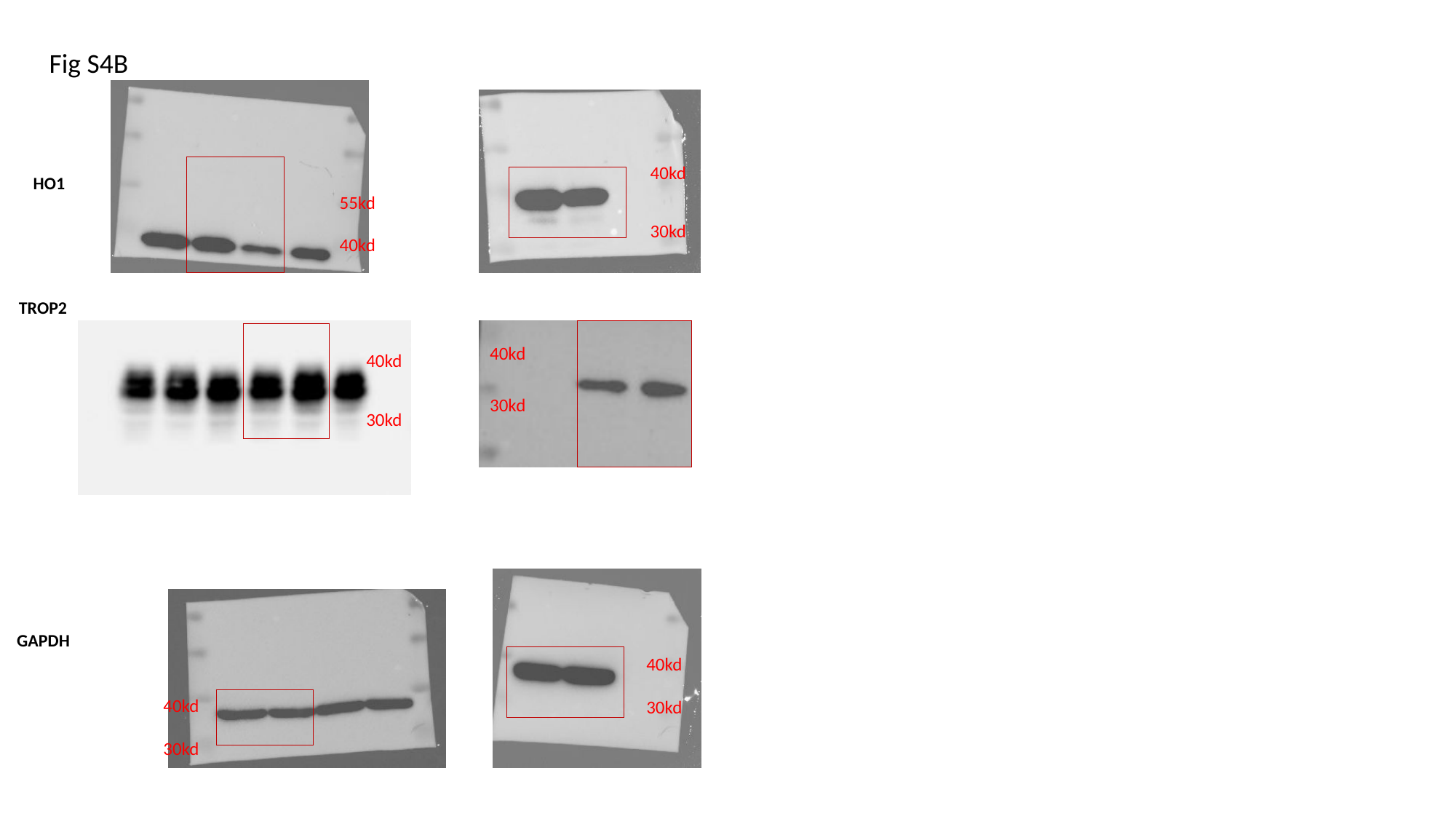

Fig S4B
40kd
HO1
55kd
30kd
40kd
TROP2
40kd
40kd
30kd
30kd
GAPDH
40kd
40kd
30kd
30kd

## Slide 10
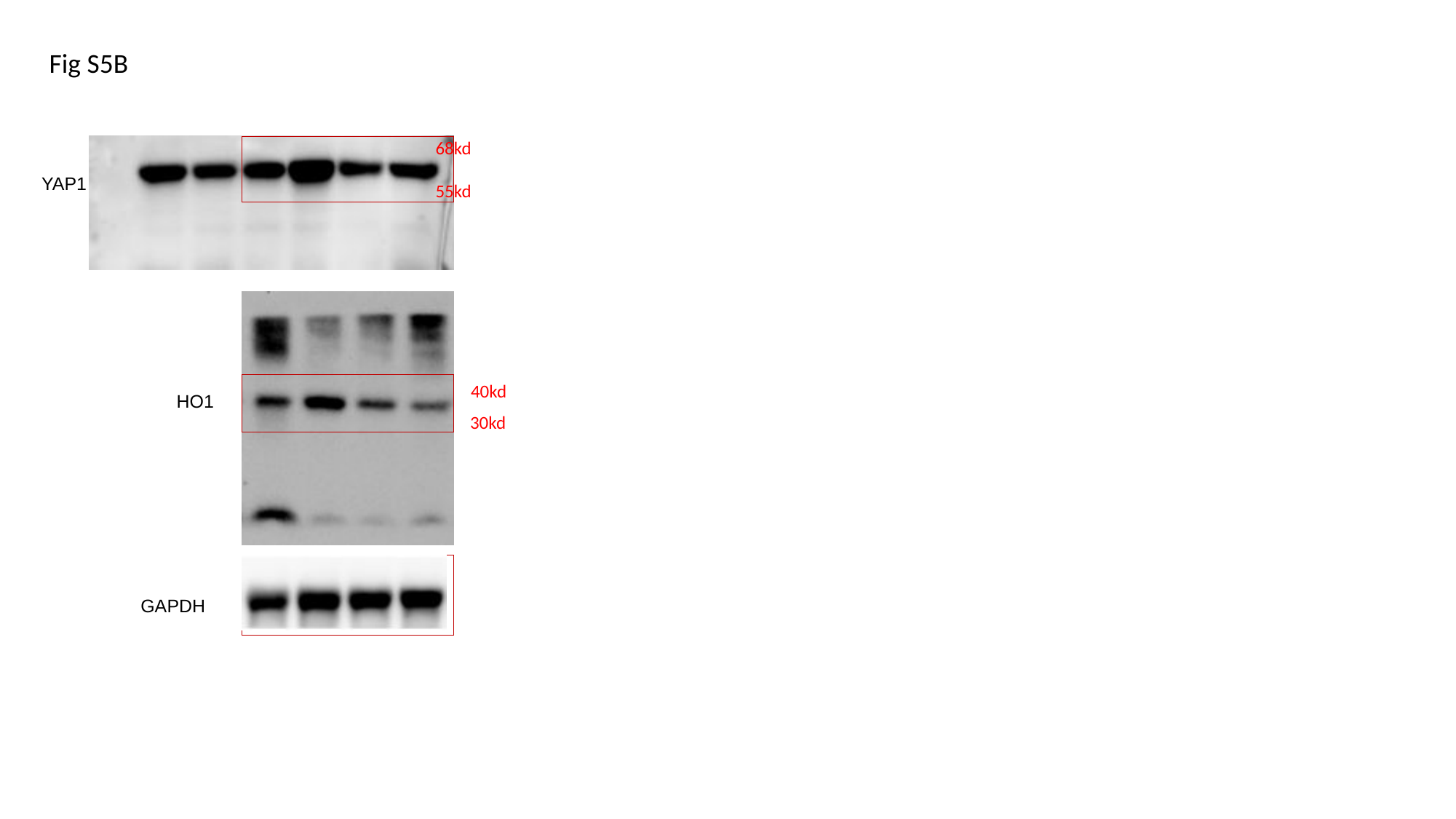

Fig S5B
68kd
YAP1
55kd
40kd
HO1
30kd
GAPDH

## Slide 11
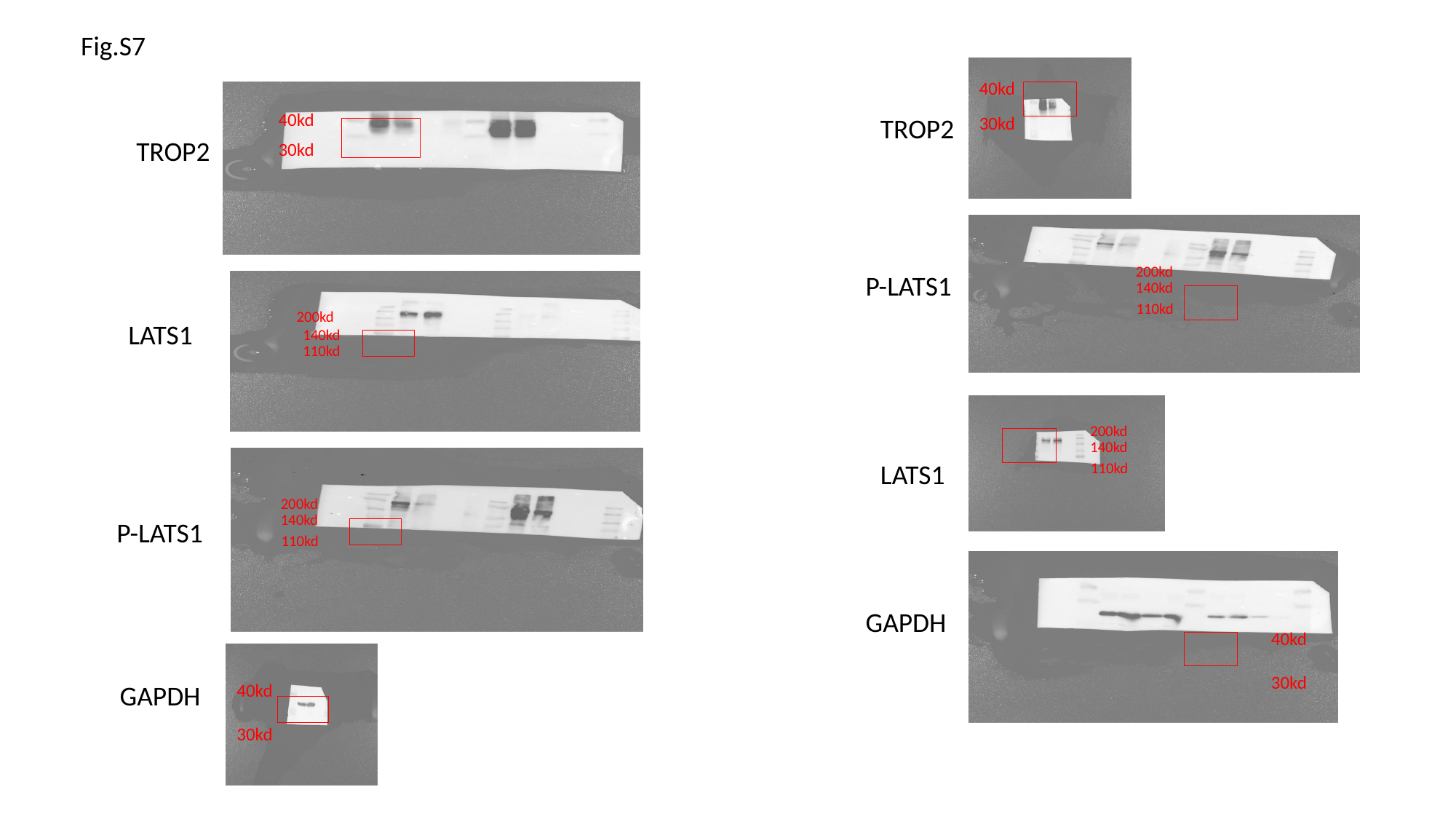

Fig.S7
40kd
40kd
TROP2
30kd
TROP2
30kd
200kd
P-LATS1
140kd
110kd
200kd
LATS1
140kd
110kd
200kd
140kd
LATS1
110kd
200kd
140kd
P-LATS1
110kd
GAPDH
40kd
30kd
GAPDH
40kd
30kd
